# EIF5A Couples Translational Control With Transcriptional Reprogramming Through Chromocenter Reorganization During Spermiogenesis

**DOI:** 10.1002/advs.202517423

**Published:** 2026-01-04

**Authors:** Yuling Cai, Tongtong Li, Qian Fang, Ziyou Bao, Feng Kong, Huitao Qi, Hanzhen Li, Mingyu Zhang, Wei Wang, Yongxia Guan, Wenbo Liu, Xiangfeng Chen, Zi‐Jiang Chen, Xiaohua Jiang, Xin Wang, Hongbin Liu

**Affiliations:** ^1^ State Key Laboratory of Reproductive Medicine and Offspring Health Center for Reproductive Medicine Institute of Women Children and Reproductive Health Shandong University Jinan 250012 China; ^2^ National Research Center for Assisted Reproductive Technology and Reproductive Genetics Shandong University Jinan Shandong 250012 China; ^3^ Key Laboratory of Reproductive Endocrinology (Shandong University) Ministry of Education Jinan Shandong 250012 China; ^4^ Shandong Technology Innovation Center for Reproductive Health Jinan Shandong 250012 China; ^5^ Research Unit of Gametogenesis and Health of ART‐Offspring Chinese Academy of Medical Sciences (No.2021RU001) Jinan Shandong 250012 China; ^6^ Suzhou Research Institute Shandong University Suzhou Jiangsu 215123 China; ^7^ Center for Stem Cell Biology and Regenerative Medicine MOE Key Laboratory of Bioinformatics School of Life Sciences Tsinghua University Beijing 100084 China; ^8^ Key Laboratory of Systems Health Science of Zhejiang Province School of Life Science Hangzhou Institute for Advanced Study University of Chinese Academy of Sciences Hangzhou 310024 China; ^9^ State Key Laboratory of Integration and Innovation of Classic Formula and Modern Chinese Medicine Linyi Shandong 276005 China; ^10^ Shanghai Key Laboratory for Assisted Reproduction and Reproductive Genetics Shanghai 200000 China; ^11^ Department of Reproductive Medicine Ren Ji Hospital Shanghai Jiao Tong University School of Medicine Shanghai 200000 China; ^12^ Center for Reproduction and Genetics, Department of Obstetrics and Gynecology The First Affiliated Hospital of USTC Division of Life Sciences and Medicine University of Science and Technology of China Hefei Anhui 230001 China; ^13^ CUHK‐SDU Joint Laboratory on Reproductive Genetics School of Biomedical Sciences The Chinese University of Hong Kong Hong Kong 999077 China

**Keywords:** chromatin accessibility, eIF5A, male infertility, spermatogenesis, translational‐transcriptional coupling

## Abstract

Eukaryotic translation initiation factor 5A (eIF5A) facilitates protein synthesis and impacts diverse biological processes, yet its role in transcriptional regulation is poorly understood. Here eIF5A highly expressed in diverse spermatogenic cell types are found. Conditional knockout of *Eif5a* (SKO) causes complete infertility in male mice due to round spermatid arrest. Interestingly, eIF5A deletion severely compromises chromocenter integrity in round spermatids. Proteomic profiling reveals widespread dysregulation in eIF5A‐deficient round spermatids, downregulated proteins are enriched for chromatin‐associated functions, likely contributing to chromocenter dysfunction. Notably, ATAC‐seq (Assay for Transposase‐Accessible Chromatin with high‐throughput sequencing) analysis shows increased chromatin accessibility upon eIF5A depletion, accompanied by transcriptional dysregulation of genes critical for acrosome and manchette formation. This data underscore that eIF5A not only regulates the translation of chromatin‐organizing proteins required for chromocenter stability but also influences transcriptional regulation by modulating chromatin landscape. These findings illuminate a previously uncharacterized and germ cell‐specific pathway coupling translational control and transcriptional regulation via chromatin reorganization.

## Graphical abstract

The translation factor Eukaryotic translation initiation factor 5A (eIF5A) is essential for male fertility in mice. It supports the translation of proteins crucial for heterochromatin organization and acrosome formation. eIF5A deficiency disrupts chromocenter integrity, increases chromatin accessibility, and causes transcriptional dysregulation, leading to defects in acrosome and manchette formation that arrest spermatid development.

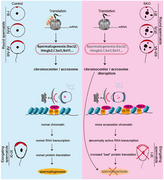

## 1 Introduction

Gene expression, a tightly regulated process, involves two fundamental steps: transcription and translation. While transcription converts DNA into messenger RNA (mRNA), translation deciphers the mRNA template to synthesize functional proteins. These processes are intricately intertwined, yet the regulatory mechanisms bridging them remain incompletely understood. In particular, the role of translation factors in modulating transcription has been underexplored, despite emerging evidence suggesting their potential cross‐talk in cellular function and development. For example, the translation initiation factor eIF4E influences transcription by regulating the nuclear export of specific mRNAs, which in turn affects downstream transcriptional programs.^[^ ^1^ ^]^ Similarly, eIF2A, a key player in translation initiation, modulates transcription under stress conditions by controlling the activity of transcription factors such as ATF4.^[^ ^2^ ^]^ These findings underscore the complex interplay between translational and transcriptional regulation. Despite these advances, the molecular pathways linking translation factors to chromatin remodeling and transcriptional control remain largely uncharacterized.

Spermatogenesis in mammals is a tightly regulated process involving mitotic proliferation, meiotic division, and spermiogenesis.^[^ ^3^, ^4^ ^]^ Precise coordination of transcriptional regulation and translational control mechanisms governs this process, ensuring the correct temporal expression of gene products.^[^ ^5^ ^]^ During spermatogenesis, chromatin condensation progressively intensifies in elongating haploid spermatids, shutting down transcriptional activity entirely during late spermiogenesis.^[^ ^6^ ^]^ Consequently, mRNAs are transcribed in advance and stored translationally inactive until required—a mechanism known as the uncoupling of transcription and translation.^[^ ^7^ ^]^ Within this context, translation initiation factors play vital roles in coordinating protein synthesis at precise stages of spermatogenesis. For example, *Eif2s3y* is critical for spermatogonial self‐renewal,^[^ ^8^ ^]^ while loss of *Eif4e‐1* or *Eif4e‐3* disrupts nuclear condensation and spermatid differentiation.^[^ ^9^ ^]^

The eukaryotic translation initiation factor eIF5A, uniquely modified by hypusine, is highly conserved across eukaryotes.^[^ ^10^, ^11^, ^12^ ^]^ Its hypusination, catalyzed sequentially by deoxyhypusine synthase (DHPS) and deoxyhypusine hydroxylase (DOHH) using spermidine, is vital for development, as evidenced by embryonic lethality upon homozygous deletion of *Eif5a*, *Dhps*, or *Dohh* and neurodevelopmental disorders linked to their variants.^[^ ^13^, ^14^ ^]^ Functionally, eIF5A facilitates polyproline motif translation, regulates elongation and termination of translation,^[^ ^15^, ^16^ ^]^ and modulates protein synthesis impacting cellular homeostasis (e.g., autophagy) and is implicated in human diseases like cancer and diabetes.^[^ ^17^, ^18^ ^]^ Nonetheless, its function within the context of germ cell development, particularly spermatogenesis, has yet to be elucidated.

Spermatogenesis involves continuous and dynamic reorganization of germ cell nuclei, beginning with mitotic spermatogonia and progressing through meiotic divisions to the formation of mature sperm.^[^ ^19^ ^]^ During this process, spermatids undergo a nuclear transformation characterized by heterochromatin reorganization.^[^ ^20^, ^21^ ^]^ Specifically, constitutive heterochromatin clusters from multiple chromosomes aggregate to form a single, dense chromocenter located centrally in the haploid nucleus, from the round spermatid stage onward.^[^ ^22^ ^]^ Disruption of this essential chromocenter structure is a hallmark of abnormalities in spermatogenesis and male infertility.^[^ ^23^, ^24^, ^25^, ^26^ ^]^ However, the regulatory mechanisms governing chromocenter formation and maintenance poorly understood.

Here, we demonstrate that eIF5A is required for spermatogenesis by specifically modulating the translation of proteins essential for maintaining chromocenter integrity in round spermatids. Disruption of *Eif5a* in mice impaired chromocenter formation, and comprehensive multi‐omics analysis (including ATAC‐seq, Smart‐seq2, and proteomic profiling) revealed that this structural defect resulted in increased chromatin accessibility. This aberrant chromatin state triggered widespread dysregulation of gene transcription and protein expression, ultimately impairing crucial processes like acrosome and manchette formation and causing male infertility.

## 2 Results

### 2.1 eIF5A is Highly Expressed in Spermatogenic Cells

**Figure 1:**
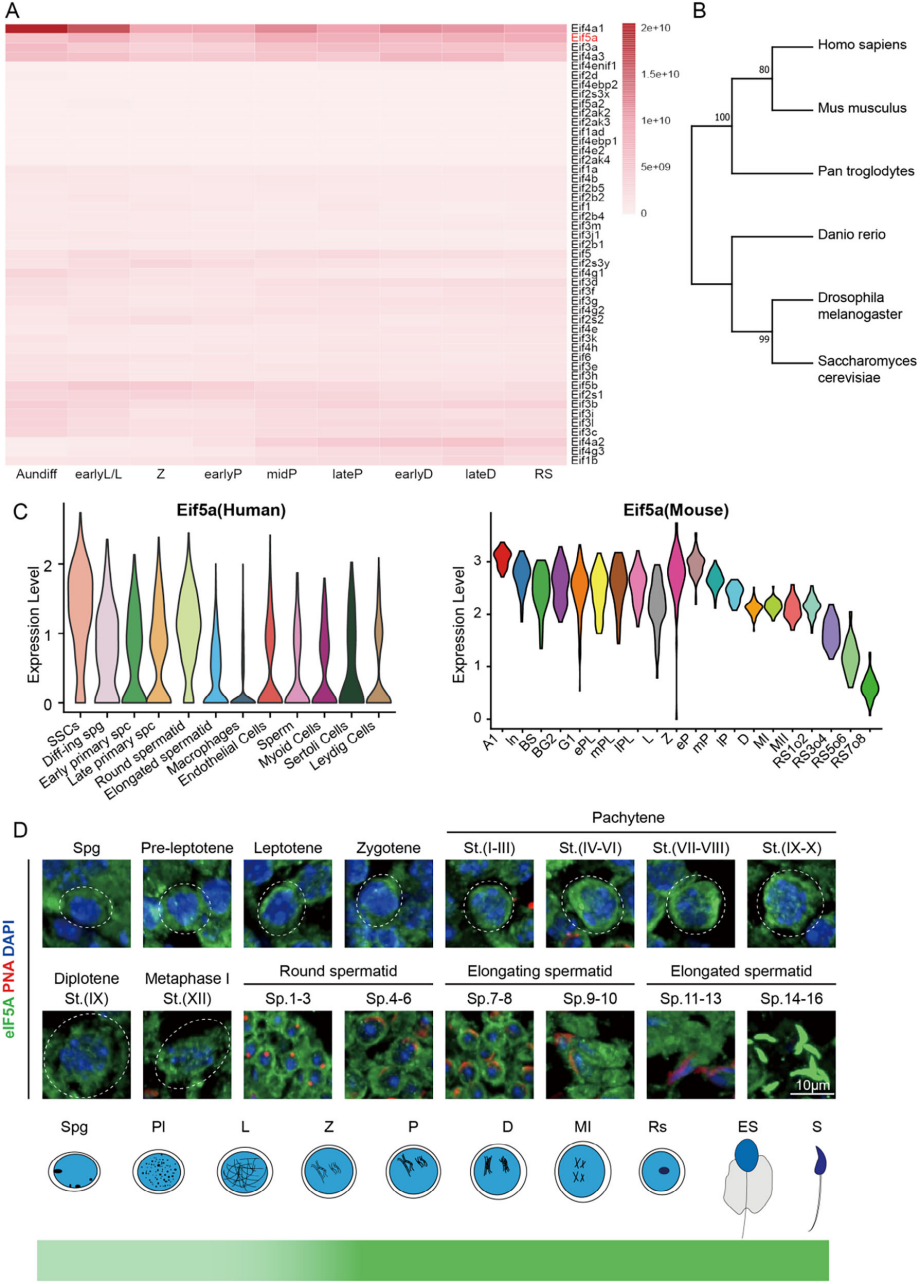
EIF5A is evolutionarily conserved and highly expressed in spermatogenic cells. A) Heatmap analysis of protein expression levels of known translation initiation factors during mouse spermatogenesis. B) Phylogenetic analysis of eIF5A based on amino acid sequences from *Homo sapiens, Mus musculus, Drosophila melanogaster, Saccharomyces cerevisiae, Danio rerio*, and *Pan troglodytes*. C).Violin plots showing *Eif5a* mRNA levels in each cell cluster identified in human (left) and mouse (right) testes by scRNA‐seq analyses. D). Co‐immunostaining for eIF5A and peanut agglutinin (PNA) in testis sections containing germ cells of wild‐type mice across the indicated stages of spermatogenesis. DNA was counterstained with DAPI(top). Scale bar, 10 µm. Spg, Spermatogonia, Pl, preleptotene, L, leptotene, Z, zygotene, eP, early pachytene, P, pachytene, D, diplotene, Rs, round spermatids, Es, elongating spermatids, S, Spermatozoa.

To investigate the crosstalk between transcription and translation during spermatogenesis, we focused on functional active translation initiation factors. Using a proteomics screen to profile eukaryotic initiation factors (eIFs) expression across different stages of mouse spermatogenesis (spermatogonia to spermatids).^[^ ^27^ ^]^ We identified eIF4A1 and eIF5A as the most highly abundant (**Figure** **1**A). Literature reports indicate that eIF4A1, an RNA helicase crucial for preventing cytosolic RNA condensation, accumulates to cellular concentrations ≈10‐fold higher than other translation factors.^[^ ^28^, ^29^ ^]^ In contrast, eIF5A is implicated in regulating cell cycle progression and differentiation by promoting the translation of specific proteins required for cellular function and structure.^[^ ^30^ ^]^ Given its established developmental roles, particularly in regulating polyProformin translation in *Saccharomyces cerevisiae*,^[^ ^31^ ^]^ we selected eIF5A for further study. Amino acid sequence alignment and phylogenetic analysis confirmed high conservation of eIF5A from *Saccharomyces cerevisiae* to *Homo sapiens* (Figure 1B).

Analysis of public human testis single‐cell RNA sequencing (scRNA‐seq) data (NCBI GEO: GSE112013) showed predominant *EIF5A* transcription in spermatogenic cells, particularly spermatogonia and round spermatids, with lower levels in somatic cells such as Sertoli and Leydig cells (Figure 1C, left). Similarly, examination of published mouse scRNA‐seq data^[^ ^32^ ^]^ demonstrate relatively high *Eif5a* expression in spermatogonia, spermatocytes, and round spermatids (Figure 1C, right). Immunofluorescence staining of paraffin‐embedded adult mouse testis sections localized eIF5A predominantly to the cytoplasm of germ cells throughout spermatogenesis, with elevated signal intensity observed in spermatocytes and round spermatids (Figure 1D). These results establish that eIF5A accumulates primarily in the cytoplasm of developing germ cells.

### 2.2 eIF5A is Essential for Male Fertility in Mice

To explore the physiological role of eIF5A in male fertility and spermatogenesis, we first assessed the genetic requirement for *Eif5a*. Given that *Eif5a*‐null mice exhibit early embryonic lethality,^[^ ^33^ ^]^ we employed a conditional knockout strategy. Using CRISPR/Cas9, we generated *Eif5a*‐floxed mice, in which exons 3 to exons 7 were flanked by loxP sites (Figure S1A, Supporting Information). To achieve germ cell–specific deletion, we crossed *Eif5a*‐floxed mice with *Stra8‐GFPCre* knock‐in mice, which express Cre recombinase in male germ cells starting from postnatal day 3 (P3), yielding *Eif5a^fl/Δ^*;*Stra8‐GFPCre* mice (referred to as SKO). PCR‐based genotyping confirmed the presence of heterozygous *Eif5a*‐floxed and homozygous SKO alleles (Figure S1B, Supporting Information). Western blots analysis verified a significant reduction in eIF5A protein levels in SKO testes compared to *Eif5a^fl/+^* control testes (hereafter termed control) (Figure S1C, Supporting Information). Immunofluorescence staining further confirmed markedly reduced eIF5A signal intensity in SKO testes (Figure S1D, Supporting Information), validating germ cell‐specific knockout.

**Figure 2:**
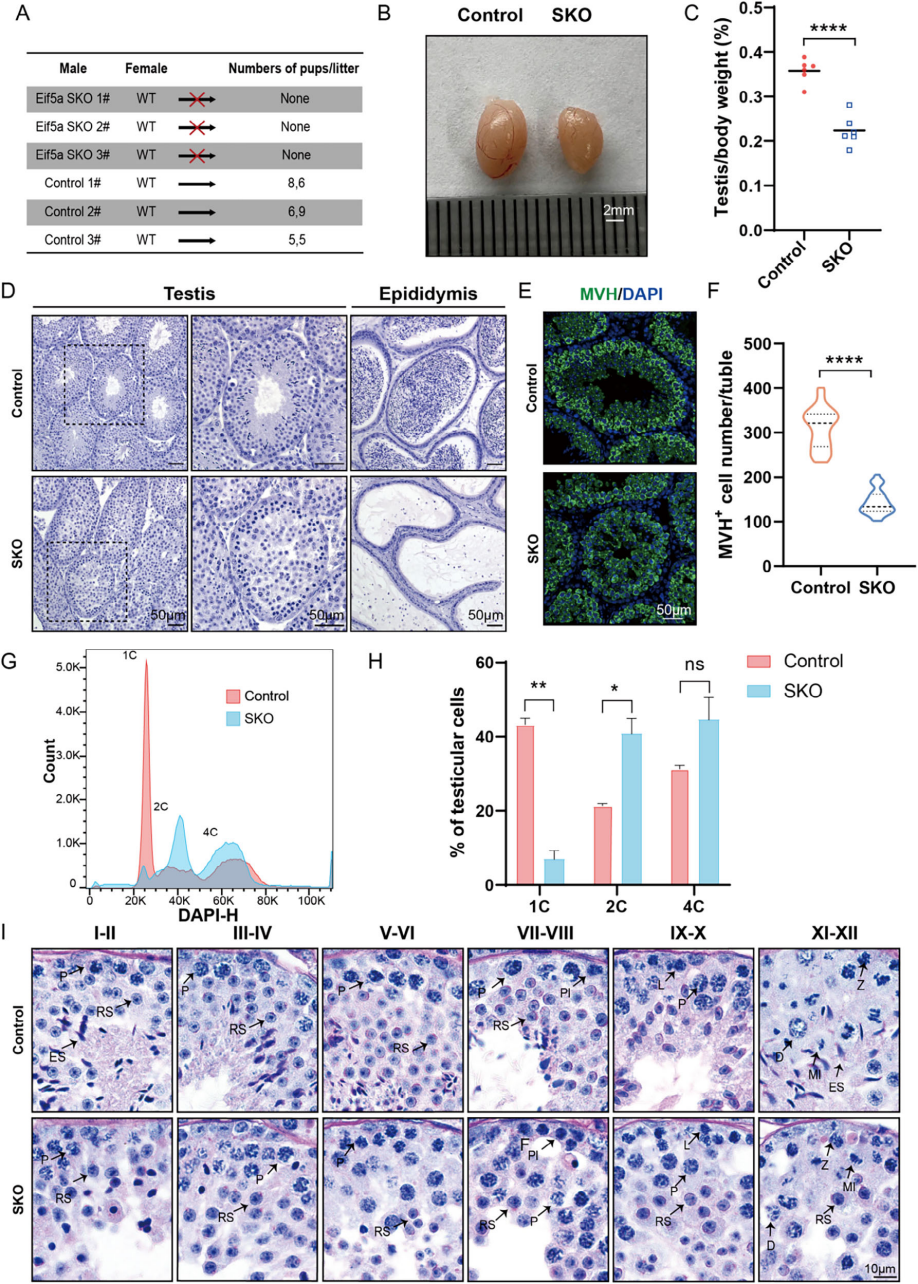
Male sterility and spermatogenic arrest at the late round spermatid stage in *Eif5a*‐deficient mice. A). Number of pups per litter from male mice (>8‐week‐old) naturally crossed with wild‐type female mice for 6 months. *Eif5a* SKO male mice were completely infertile. B) Gross morphology of representative testes from control and *Eif5a* SKO mice. Scale bar, 2 mm. C). Testes weight: body weight ratios in *Eif5a* SKO and age‐matched control mice. Data are presented as mean ± SEM, n = 6. ∗∗p < 0.01 by two‐tailed unpaired Student's *t*‐test. D).Hematoxylin staining in testes and epididymis sections from 12‐week‐old control and *Eif5a* SKO mice. Scale bar, 50 µm. E).Immunofluorescence staining for the VASA homolog, MVH, in testes of 12‐week‐old control and *Eif5a* SKO mice. Scale bar: 50 µm. F).MVH‐positive cell counts per tubule in the varicocele of control and *Eif5a* SKO mice, n = 3 mice/genotype, P<0.0001 by mice/genotype by two‐tailed unpaired Student's *t*‐test. G).Representative FACS profiles from Hoechst‐stained testicular cells of an 12‐week‐old mouse. Peaks correspond to N, 2N, and 4N cell populations. H).Quantitative analysis of control and *Eif5a* SKO germ cell subpopulations (n = 3 mice/genotype). Data are expressed as a percentage (%) of germ cells and show mean ± SEM, **p < 0.01 by two‐tailed unpaired Student's *t*‐test. I). PAS‐hematoxylin staining of testis sections from 12‐week‐old control and *Eif5a* SKO mice. Stages of seminiferous epithelium cycles were determined by the morphology of spermatocytes and round spermatids. Pl, preleptotene, L, leptotene, Z, zygotene, P, pachytene, D, diplotene, RS, round spermatids, ES, elongating spermatids. Scale bar, 10 µm.

To evaluate the impact of conditional *Eif5a* knockout on male fertility, two‐month‐old wild‐type (WT) females were continuously housed for six months with control or SKO males. None of the three SKO males produced litters during this period, while control males sired litters averaging ≈13 pups per male (**Figure** **2**A). SKO mice also exhibited significantly smaller testes (Figure 2B), with a testis‐to‐body weight ratio ∼30% lower than controls (Figure 2C). Collectively, these results demonstrate that eIF5A is indispensable for male fertility in mice and plays a critical role in spermatogenesis.

### 2.3 Haploid Germ Cell Development is Defective in SKO Mice

To further investigate the role of eIF5A in spermatogenesis, we performed hematoxylin analysis of testicular and epididymal sections from 8‐week‐old control and SKO mice. Histological examination revealed impaired spermatogenesis in SKO males (Figure 2D). Specifically, no mature spermatozoa were observed in the epididymal lumen, and elongated spermatids were largely absent within the testes of SKO mice (Figure 2D). Immunofluorescence (IF) staining for germ cell marker MVH indicated a significant loss of germ cells in SKO testes (Figure 2E,F). Further supporting this finding, fluorescence‐activated cell sorting (FACS) analysis demonstrated a marked reduction in the proportion of haploid spermatids within SKO testes (Figure 2G, Figure S2A, Supporting Information). Quantification of DNA content by flow cytometry revealed a dramatic decrease in haploid spermatids, from ≈45% in control testes to ≈8% in SKO testes (Figure 2H).

To identify the stage at which spermatogenesis is arrested, we first assessed meiotic progression. Chromosome spread analysis from 3‐week‐old control and SKO mice, immunostained for synaptonemal complex proteins SYCP1 (central element) and SYCP3 (lateral element),^[^ ^34^, ^35^ ^]^ showed no significant differences in the distribution patterns of these proteins across meiotic prophase I substages (Figure S2B, Supporting Information). Quantification of cells at defined stages—leptotene, zygotene, pachytene, diplotene, and metaphase I—showed comparable frequencies between genotypes (Figure S2C, Supporting Information), indicating normal meiotic progression in the absence of eIF5A. Additionally, staining for the DNA double‐stranded break (DSB) marker γH2AX revealed no apparent defects in meiotic DSB repair in SKO mice (Figure S2D, Supporting Information).

To investigate post‐meiotic development, testicular sections from adult mice were analyzed using hematoxylin and Periodic Acid‐Schiff (PAS) staining. This examination indicated that spermatogenesis was arrested at step 8 spermatid stage in adult SKO testes, characterized by the absence of elongating spermatids (Figure 2I). TUNEL assays further revealed prominent apoptotic signals specifically within the seminiferous tubules of SKO mice, indicating enhanced germ cell death (Figure S2E,F, Supporting Information). Collectively, these observations suggest that spermatogenesis in SKO mice arrests after meiosis, with critical defects occurring during haploid germ cell development.

### 2.4 EIF5A Deficiency Disrupts Acrosome and Manchette Formation

**Figure 3:**
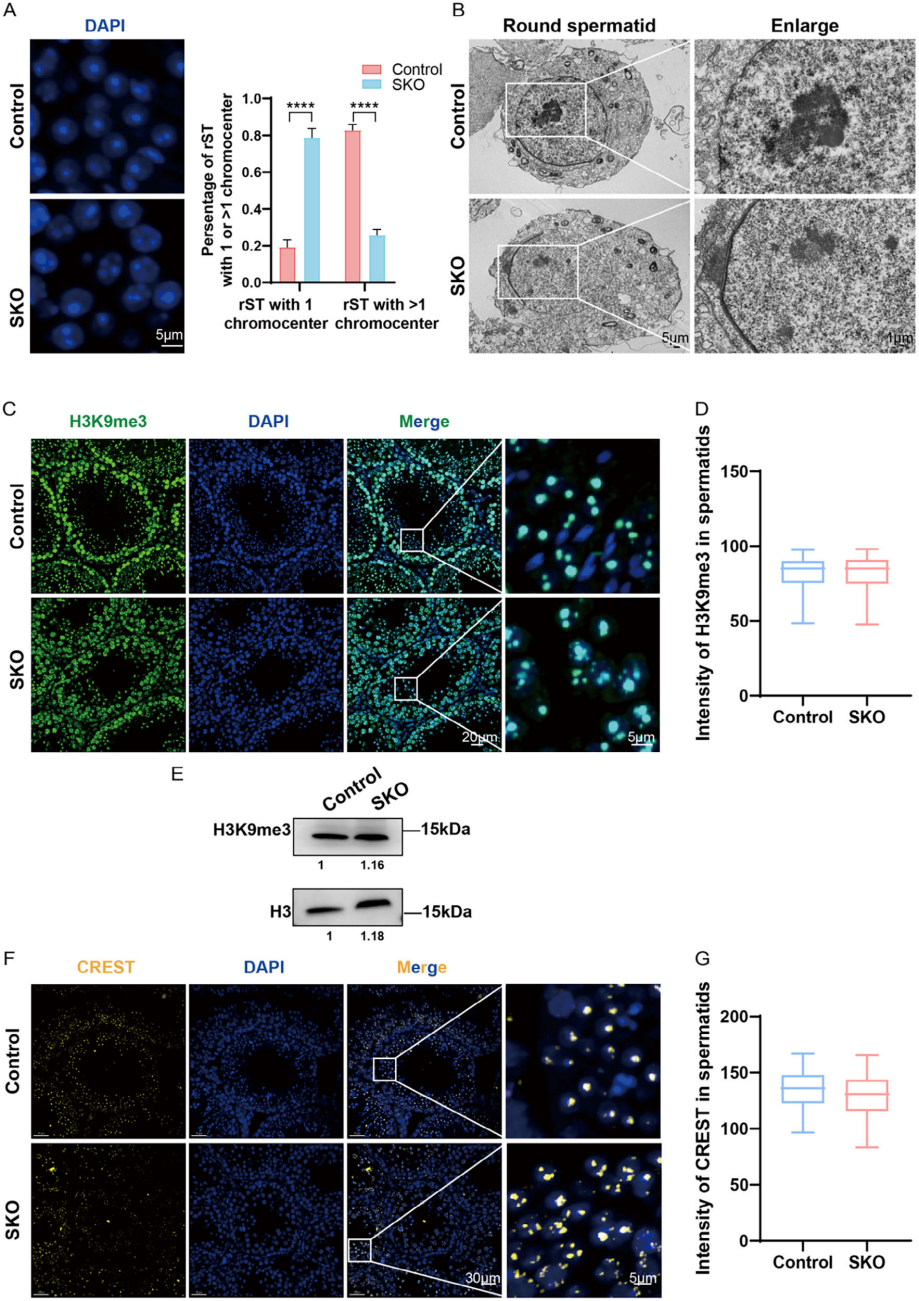
Severe chromocenter fragmentation in round spermatids of *Eif5a* SKO mice. A).DAPI staining in control and Eif5a SKO round spermatids (left, Scale bars: 5 µm) and the quantitative results (right). Data are presented as mean ± SD (n = 3 mice/genotype). ****P < 0.0001 (two‐tailed unpaired t‐test). B). Representative transmission electron micrographs of round spermatid nuclear ultrastructure in 10‐week‐old control and *Eif5a* SKO mice. Scale bars, 5 µm (left), 1 µm (right). C).Co‐staining for H3K9me3 (green) and DNA (blue) in testicular sections from 12‐week‐old *Eif5a* SKO and control mice. Scale bars, 20 µm (left and middle), 5 µm (far right). D) Quantification of H3K9me3 fluorescence intensity from panel C. Data are presented as mean ± SEM,n = 3 mice/genotype. No statistically significant difference was found as determined by two‐tailed unpaired Student's *t*‐test (n.s.). E) Western blots of H3K9me3 in testes from 12‐week‐old control and *Eif5a* SKO testes. Histone3 H3 served as the loading control. F).Co‐staining for CREST (yellow) and DNA (blue) in testicular sections of 12‐week‐old *Eif5a* SKO and control mice. Scale bars,30 µm (left), 5 µm (right). G).Quantification of CREST fluorescence intensity from panel F. Data are presented as mean ± SEM,n = 3 mice/genotype. No statistically significant difference was found as determined by two‐tailed unpaired Student's *t*‐test (n.s.).

To better understand how eIF5A contributes to post‐meiotic spermatid development, we analyzed the formation of key spermiogenic structures: the acrosome and the manchette. Acrosome biogenesis comprises four sequential stages: the Golgi phase, the cap phase, the acrosome phase, and the maturation phase.^[^ ^36^ ^]^ We examined acrosomal morphology using IF staining for peanut agglutinin (PNA), a marker for the outer acrosomal membrane (Figure S3A, Supporting Information). In control testes, spermatids displayed characteristic PNA staining patterns corresponding to the four stages: nascent acrosomal vesicles derived from proacrosomal granules (Golgi phase, steps 1–3), flattened vesicle formation (cap phase, steps 4–6), thin‐layered acrosomal vesicle (steps 7–8), and nuclear encapsulation by mature acrosome (acrosomal phase, steps 9–16) (Figure S3A, Supporting Information). In contrast, SKO round spermatids exhibited irregular distribution. Critically, they failed to form a cohesive acrosomal vesicle during the Golgi phase (Figure S3A, Supporting Information). This defect persisted into the cap phase, where PNA signal appeared as punctate foci along the nuclear membrane rather than forming a distinct cap‐like structure (Figure S3A, Supporting Information). Transmission electron microscopy (TEM) analysis further validated the acrosomal defects. Compared to control spermatids exhibiting clear acrosomal structures in the Golgi, cap, and acrosomal phases (Figure 3B), SKO round spermatids showed disorganized acrosomal systems. During the Golgi phase, electron‐dense acrosomal granules localized peripherally rather centrally within the nascent acrosome (Figure S3B, Supporting Information). In stage II‐III SKO round spermatids, acrosomal granules failed to fuse and were distributed in the cytoplasm, accumulating near the outer nuclear membrane (Figure S3B, Supporting Information). Collectively, these results demonstrate that eIF5A deficiency disrupts acrosomal biogenesis starting at the Golgi phase, suggesting that this translation initiation factor is essential for proper acrosome formation during spermatogenesis.

To assess manchette formation, we performed immunostaining for α‐tubulin, a component of this perinuclear microtubule array. Control round spermatids displayed the characteristic manchette morphology. In contrast, SKO round spermatids exhibited a collapsed and disorganized α‐tubulin structure, indicating that loss of eIF5A disrupts manchette assembly (Figure S3C, Supporting Information). These observations suggest that eIF5A is indispensable for the structural integrity and formation of both the acrosome and the manchette during spermiogenesis.

### 2.5 EIF5A Deficiency Induces Chromocenter Fragmentation in Round Spermatids

During histological examination of SKO testes, we unexpectedly noted an aberrant nuclear phenotype in round spermatids. Unlike control spermatids, which typically display a single prominent chromocenter (marked by intense hematoxylin staining), SKO round spermatids consistently exhibited multiple chromocenters (Figure 2I). Chromocenters represent dense heterochromatin foci crucial for nuclear organization in post‐meiotic round spermatids,^[^ ^37^ ^]^ with a single chromocenter being a defining feature.

To characterize this defect, we performed DAPI staining, which confirmed the presence of multiple fragmented chromocenters in the nuclei of SKO round spermatids (**Figure** **3**A). TEM analysis corroborated these findings: nuclei of SKO round spermatids contained multiple discrete dense heterochromatin regions, contrasting with the single, large chromocenter observed in control round spermatids (Figure 3B). IF staining and Western blot analysis for H3K9me3, a marker of constitutive heterochromatin, revealed comparable protein levels between control and SKO samples (Figure 3C–E). However, their subnuclear distribution differed markedly. While H3K9me3 localized exclusively to a single distinct region corresponding to the chromocenter in controls, SKO round spermatids consistently exhibited two or more H3K9me3 foci (Figure 3C).

Moreover, we assessed centromere organization using antibodies from CREST patient serum. These antibodies target centromeric proteins, which typically aggregate into a single chromocenter during spermatogenesis.^[^ ^38^ ^]^ Consistent with chromocenter fragmentation, SKO round spermatids exhibited multiple dispersed CREST‐positive foci, whereas control round spermatids presented a single large CREST‐positive cluster (Figure 3F). Importantly, the total CREST fluorescence intensity per nucleus was comparable between control and SKO spermatids, demonstrating that the centromeric material was redistributed rather than increased (Figure 3G). These data suggest that loss of eIF5A disrupts normal chromocenter formation and chromatin organization during spermatid differentiation, leading to chromocenter fragmentation.

### 2.6 eIF5A Promotes Translational Elongation in Round Spermatids

**Figure 4:**
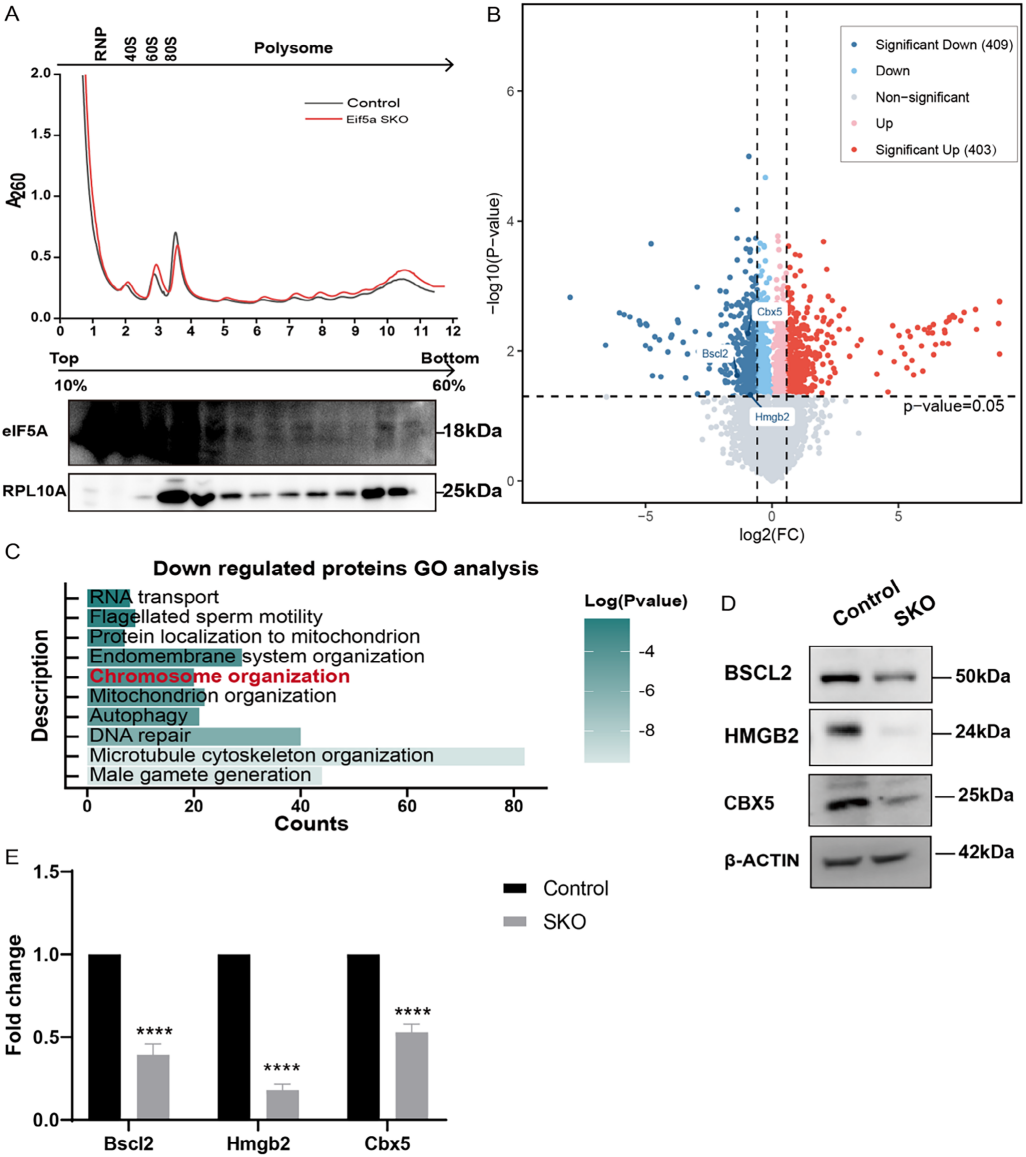
Reduced translation of spermatogenesis‐related proteins in round spermatids of *Eif5a* SKO mice. A).Absorbance profile (OD = 260 nm) of sucrose gradient sedimentation of cell extracts from control and *Eif5a* SKO testes. Gradients were collected in 12 fractions, starting from the lightest fractions (top), Western blot analysis of eIF5A and RPL10A distribution in each fraction of the gradient (bottom). B) Volcano plot of differential protein levels in 12‐week‐old *Eif5a* SKO round spermatids versus controls. Average log_2_ of fold change in protein abundance (n = 2 replicates) were plotted against log10 of p‐values between groups determined by t‐test. Criteria for significant differential protein abundance were p ≤0.05 and |1.5|‐fold change in *Eif5a* SKO versus control round spermatids. C).Gene ontology (GO) analysis of biological processes (BP) enriched in down‐regulated proteins of *Eif5a* SKO round spermatids (adjusted *P* < 0.05, FC<0.67). D).Western blots show BSCL2, HMGB2, CBX5, and SIRT1 proteins in *Eif5a* SKO and control mice. β‐Actin served as the loading control. E). Translation rates of *Bscl2, Hmgb2* and *Cbx5* mRNAs at translation initiation sites. Data are presented as mean ± SD. Significance was determined by two‐tailed unpaired Student's *t*‐test with n = 3 biological replicates. **p < 0.01, ****p < 0.0001.

To elucidate the mechanism underlying the impaired chromocenter formation, we investigated the impact of eIF5A on translation, given its established role as a translation factor. We first examined polysome profiles from round spermatids. Sucrose density gradient centrifugation revealed that eIF5A co‐fractionated strongly with 40S/60S monosomal complexes, 80S ribosomes, and polysomes (**Figure** **4**A). This association with actively translating ribosomes is consistent with its proposed function in regulating translation elongation.^[^ ^39^ ^]^

To directly assess the effects of eIF5A deletion on translation elongation dynamics, we performed Ribo‐seq on FACS‐isolated round spermatids from control and SKO mice at postnatal day 28 (primarily step 8 stage). Quality assessment demonstrated high‐quality data: the ribosome‐protected fragments (RPFs) exhibited the expected size distribution (29 nt) and strong 3‐nt periodicity across coding sequences (CDS), with predominant phasing at the first nucleotide of codons (Figure S4A,B, Supporting Information).

Then the analysis of global ribosome occupancy revealed a distinct pattern in SKO round spermatids: increased occupancy over the initial ≈250 codons (including the start codon region) compared to controls, coupled with a reduction in occupancy across the remainder of the CDS (Figure S4C, Supporting Information). This increase was also reflected in a significant 5′ shift in global ribosome positioning along mRNAs (Figure S4D, Supporting Information).This pattern is indicative of widespread elongation defects. Given the conserved role of eIF5A in resolving polyproline (Pro)‐mediated ribosomal stalling,^[^ ^16^ ^]^ we specifically investigated ribosome occupancy at motifs containing consecutive Pro codons. Strikingly, average ribosome occupancy at di‐proline (PP) or tri‐proline (PPP) motifs was approximately 2‐fold higher in SKO round spermatids than in controls (Figure S4E,F, Supporting Information). Furthermore, SKO round spermatids displayed enhanced translational pausing at specific codons encoding aspartic acid (D), alanine (A), and serine (S) compared to controls (Figure S4G, Supporting Information). Collectively, these results indicate that eIF5A deficiency in round spermatids disrupts normal translational elongation, evidenced by the accumulation of ribosomes near transcript starts, elevated stalling at polyproline motifs, and increased pausing at specific codons. This establishes that eIF5A is essential for promoting efficient translational elongation during spermatogenesis.

### 2.7 eIF5A Deficiency Impairs Translation of Heterochromatin Organization Factors in Round Spermatids

To identify specific proteome alterations resulting from eIF5A deficiency, we isolated round spermatids from control and SKO mice by flow cytometry for low‐input proteomic sequencing (Figure S5A, Supporting Information). Principal component analysis (PCA) revealed distinct clustering between the proteomes of control and SKO spermatids, indicating significantly divergent profiles (Figure S5B, Supporting Information). While proteomic sequencing identified comparable numbers of unique proteins in both groups (control: 6680, SKO: 6703) (Figure S5C, Supporting Information), differential expression analysis identified 812 proteins with significantly altered abundance (≥|1.5|‐fold change) in SKO spermatids. Among these, 409 proteins were downregulated and 403 proteins were upregulated upon conditional knockout of *Eif5a* (Figure 4B).

Gene ontology (GO) analysis of the downregulated proteins revealed enrichment in terms including “RNA transport”, “flagellated sperm motility”, and “protein localization to mitochondrion”, suggesting potential disruption of multiple cellular processes (Figure 4C). Critically, the term “chromosome organization” was significantly enriched, indicating eIF5A deficiency might impair chromocenter assembly or maintenance (Figure 4C). Within this downregulated group, BSCL2, HMGB2, and CBX5 were of particular interest, as BSCL2 and HMGB2 are reported regulators of chromocenter formation,^[^ ^25^, ^40^ ^]^ and CBX5 is essential for chromatin compaction.^[^ ^41^ ^]^ Western blot analysis confirmed significant downregulation of these proteins in SKO round spermatids (Figure 4D). To determine whether these reductions stemmed from translational regulation, we performed Targeted Profiling of RNA Translation (TPRT).^[^ ^42^ ^]^ TPRT analysis confirmed significantly decreased translational efficiency for *Bscl2*, *Hmgb2*, and *Cbx5* mRNAs in SKO round spermatids (Figure 4E). These findings suggest that eIF5A deficiency selectively impairs the translation of key regulators critical for heterochromatin organization and chromocenter integrity during spermatogenesis.

### 2.8 EIF5A Depletion Alters Chromatin Accessibility and Drives Transcriptional Dysregulation

**Figure 5:**
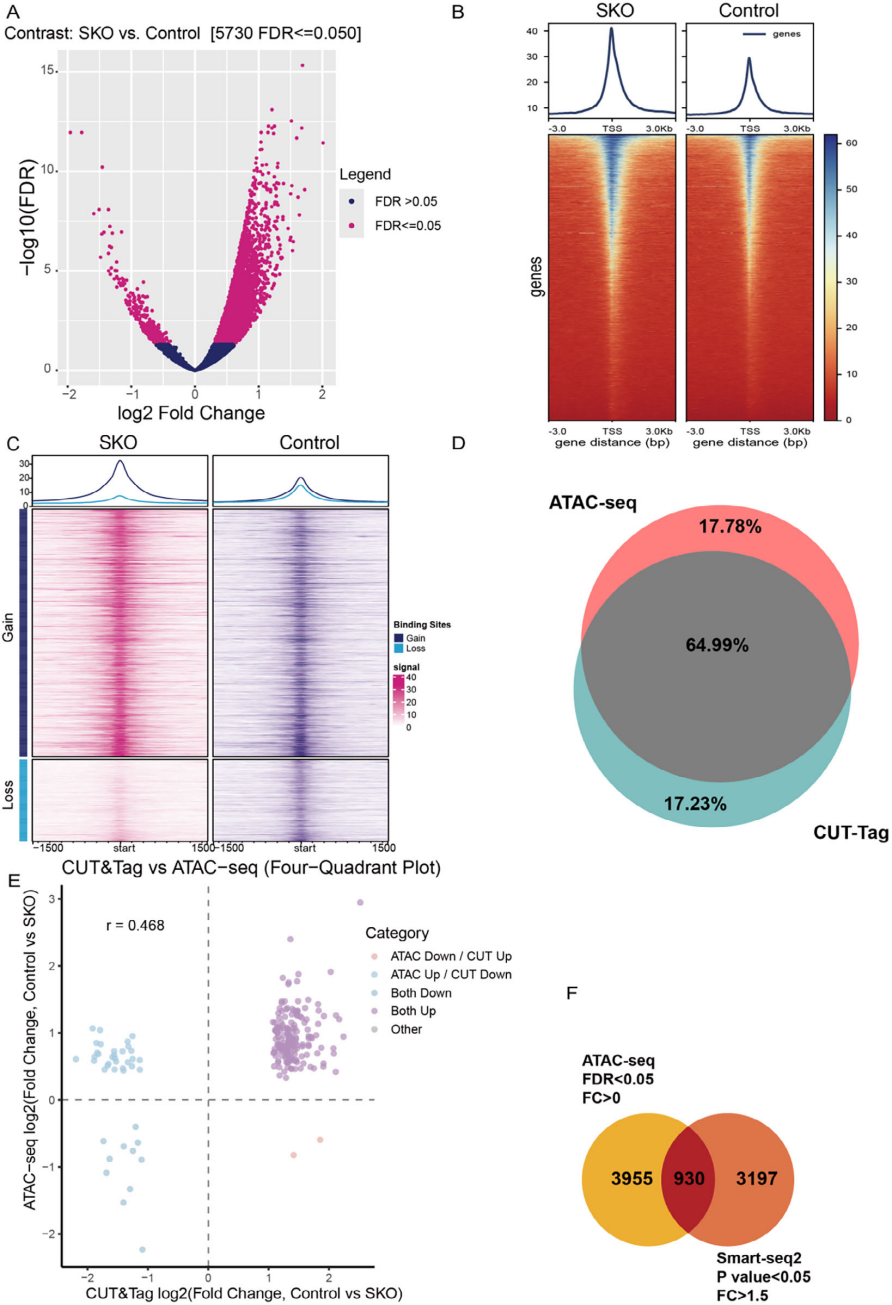
Increased chromatin accessibility in knockout round spermatids. A).Volcano plot of DEGs between control and *Eif5a* SKO samples.FDR<0.05. B.The average tag density plot(top pannel) and heatmaps (bottom pannel)around TSS (±3 kb) for the enrichment of ATAC‐seq reads in control and *Eif5a* SKO round spermatids. C).Plot shows Gain and Loss sites in all control and *Eif5a* SKO sample replicates. D).Venn diagram showing the overlap of differential peaks identified by CUT&Tag and ATAC‐seq between WT and CKO groups. E).Four‐quadrant scatter plot comparing the log_2_ fold changes of significantly differential peaks (FDR < 0.05) from H3K4me3 CUT&Tag (x‐axis) and ATAC‐seq (y‐axis). The Pearson correlation coefficient for the compared data is 0.468. Pearson's *r* = 0.468. F) Venn diagram show shared genes between ATAC‐seq (FDR<0.05) and Smart‐seq2 (FDR<0.05, FC>1.5).

Chromocenters serve as vital heterochromatin hubs for maintaining chromatin integrity and spatial nuclear organization. Their disruption in eIF5A‐deficient spermatids suggests potential global alterations in chromatin dynamics, such as shifts in compaction or accessibility, which could impact transcriptional regulation. Therefore, to assess genome‐wide chromatin accessibility changes, we performed ATAC‐seq on flow‐sorted round spermatids from control and SKO mice.^[^ ^43^ ^]^ PCA of ATAC‐seq profiles revealed clear separation between SKO and control groups along the PC1 axis, indicating distinct chromatin landscapes (Figure S6A, Supporting Information). Although the genomic distribution of accessible regions was comparable between groups (Figure S8A, Supporting Information), heatmap visualization with hierarchical clustering showed robust reproducibility within groups and clear separation between them (Figure S8B, Supporting Information). Differential peak analysis identified 5730 genomic loci with significantly altered accessibility in SKO round spermatids (FDR < 0.01) (**Figure** **5**A). Both groups showed peak enrichment at transcription start sites (TSS) (Figure 5B). Strikingly, chromatin accessibility increased at 4556 gene‐associated loci and decreased at 317 loci in SKO round spermatids (Figure 5C).

To further validate the chromatin remodeling suggested by ATAC‐seq, we performed CUT&Tag for H3K4me3, an active promoter mark. Sample correlation analysis demonstrated high reproducibility within genotypes and clear separation between WT and SKO groups (Figure S7A,C, Supporting Information). The distribution of H3K4me3 signals was strongly enriched around transcription start sites across all samples (Figure S7D, Supporting Information), consistent with its canonical role. We observed widespread alterations in H3K4me3 deposition in SKO spermatids, as visualized by a heatmap of differential binding regions (Figure S7E, Supporting Information). Importantly, integration of H3K4me3 and ATAC‐seq data revealed substantial concordance, with 64.99% of annotated targets overlapping between the two assays (Figure 5D). Correlation analysis further demonstrated a consistent positive association (Pearson r = 0.295) that strengthened (Pearson r = 0.468) when focusing on significantly differential peaks (FDR < 0.05) (Figure 5E, Figure S7F, Supporting Information). These orthogonal data strongly support that eIF5A deficiency induces genuine and coordinated chromatin remodeling.

To determine functional consequences, we integrated these accessibility changes with transcriptomes from Smart‐seq2 analysis. Replicate correlations and hierarchical clustering confirmed data quality and distinct SKO/control clustering (Figure S8A, Supporting Information). PCA of transcriptomic profiles positioned round spermatids among testicular germ cells^[^ ^32^ ^]^ (Figure S8B, Supporting Information). Integration of ATAC‐seq and Smart‐seq2 data revealed that 22.5% (n = 930) of significantly upregulated genes in SKO spermatids (Fold Change≥1.5, p value < 0.5) coincided with newly accessible chromatin region absent in controls (Figure 5F). This indicates that chromocenter disruption by eIF5A deficiency triggers widespread chromatin accessibility changes closely linked to transcriptional dysregulation.

For the remaining 77.5% of DEGs without accessibility changes, we analyzed transcription factor (TF) motifs enriched in gained ATAC‐seq peaks (Figure S6B, Supporting Information). GO analysis of corresponding TFs revealed significant enrichment for “transcription by RNA polymerase II” and “positive regulation of miRNA transcription”. This suggests TFs binding to newly accessible regions may activate transcription of downstream genes independent of local chromatin changes. These findings collectively indicate that eIF5A deficiency remodels the chromatin landscape, driving both direct accessibility‐mediated and indirect TF‐mediated transcriptional dysregulation during spermatogenesis.

### 2.9 Transcriptional Dysregulation Drives Acrosome and Manchette Defects

**Figure 6:**
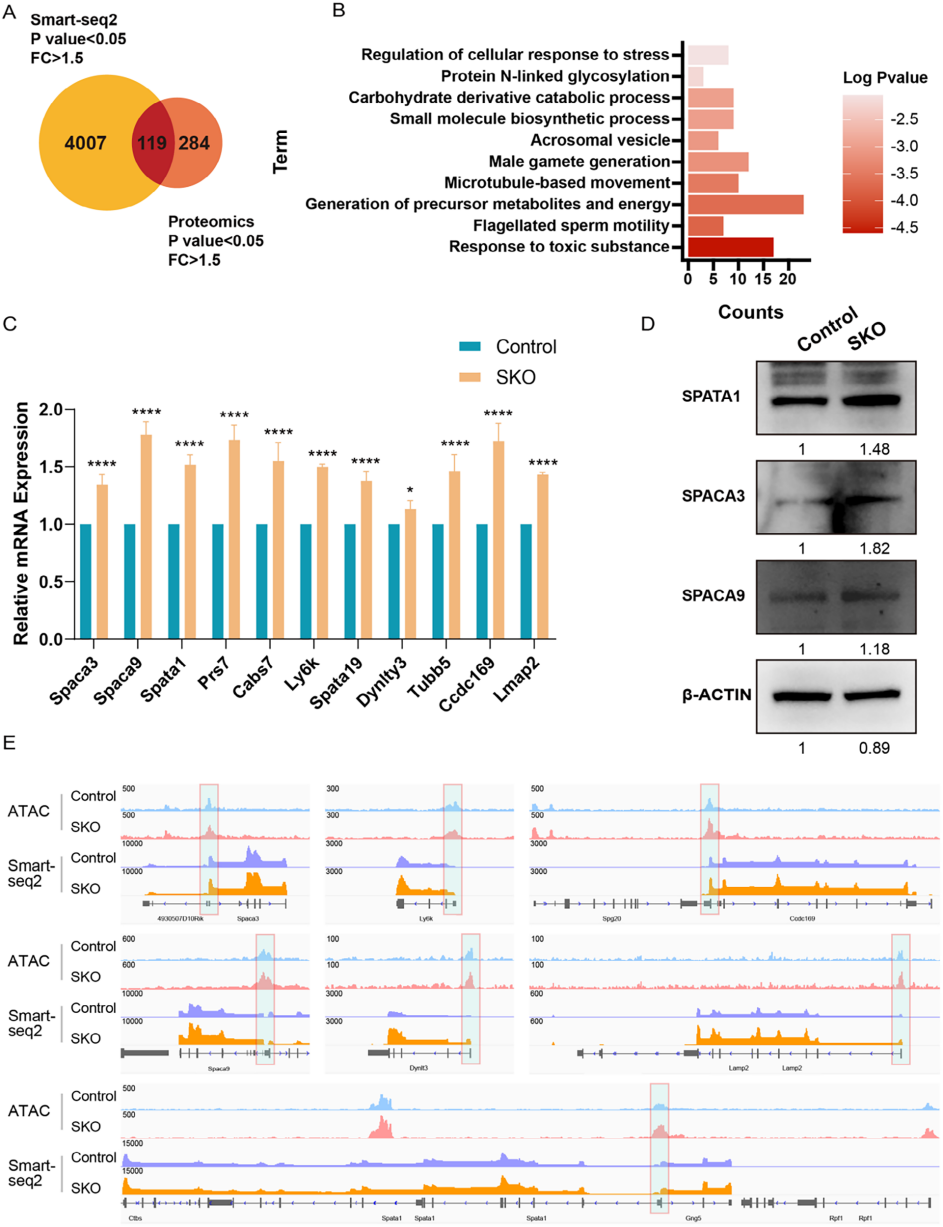
Proteomic alterations associated with transcriptional changes induced by *Eif5a* deletion. A).Venn diagram of shared genes between Smart‐seq2 (P value<0.05, FC>1.5) and Proteomics (P<0.05, FC>1.5) analyses. B).GO enrichment analysis based on the 119 commonly upregulated genes. C).QRT‐PCR analysis of candidate genes that were consistently dysregulated in both the transcriptome and proteome of *Eif5a* SKO testes. Data are presented as mean ± SD from three independent biological replicates (n = 3). Statistical significance was determined using a two‐tailed, unpaired Student's t‐test (*P < 0.05, **P < 0.01, ***P < 0.001, ****P < 0.0001). D). Western blots show SPATA1, SPACA3 and SPACA9 proteins in Eif5a SKO and control mice. β‐Actin served as the loading control. E). IGV visualization of genomic regions harboring acrosome‐related (*Spaca3, Ly6K,Spaca9,Spata1,Lamp2*) and microtubule‐associated (*Ccdc169, Dynlt3*) genes. Top: ATAC‐seq tracks showing chromatin accessibility in control (blue) versus SKO (red) round spermatids. Bottom: Corresponding Smart‐seq2 coverage.

Our proteomic analysis identified 403 proteins significantly upregulated in SKO round spermatids compared to controls. Given that eIF5A primarily functions as a translation promoting factor, this observation initially appears counterintuitive. However, considering the widespread transcriptional changes and increased chromatin accessibility observed in SKO round spermatids, we investigated whether aberrant transcription contributes to the observed protein upregulation. Analysis of Smart‐seq2 data revealed 4127 upregulated transcripts among 8711 DEGs in SKO round spermatids compared to controls (Fold Change ≥ 1.5, p value < 0.05) (Figure S8C, Supporting Information). Integrating this with the proteomic data showed that 30% of the upregulated proteins also exhibited increased mRNA levels (**Figure** **6**A). GO analysis of these concordantly upregulated genes/proteins identified significant enrichment for biological processes critical to spermatid differentiation: “acrosome formation” and “microtubules organization” (Figure 6B). To directly dissect the relationship between the alterations in chromatin accessibility and transcriptional dysregulation, we performed integrative genomic browser (IGV) analysis of representative acrosome‐ (*Spaca3, Ly6K, Spaca9, Spata1, Lamp2*) and microtubule‐associated (*Ccdc169, Dynlt3*) genes (Figure 6E). Strikingly, the chromatin accessibilities at the transcriptional initiation sites of these upregulated genes, which were confirmed by RT‐qPCR and Western blot (Figure 6C,D), significantly increased in SKO spermatids. Collectively, these findings suggest that eIF5A deficiency disrupts the precise coordination of transcription and translation. This dysregulation causes abnormal overexpression of specific acrosome‐ and microtubule‐associated genes by increasing their chromatin accessibility, ultimately contributing to the structural defects in the acrosome and manchette during spermatogenesis.

## 3 Discussion

**Figure 7:**
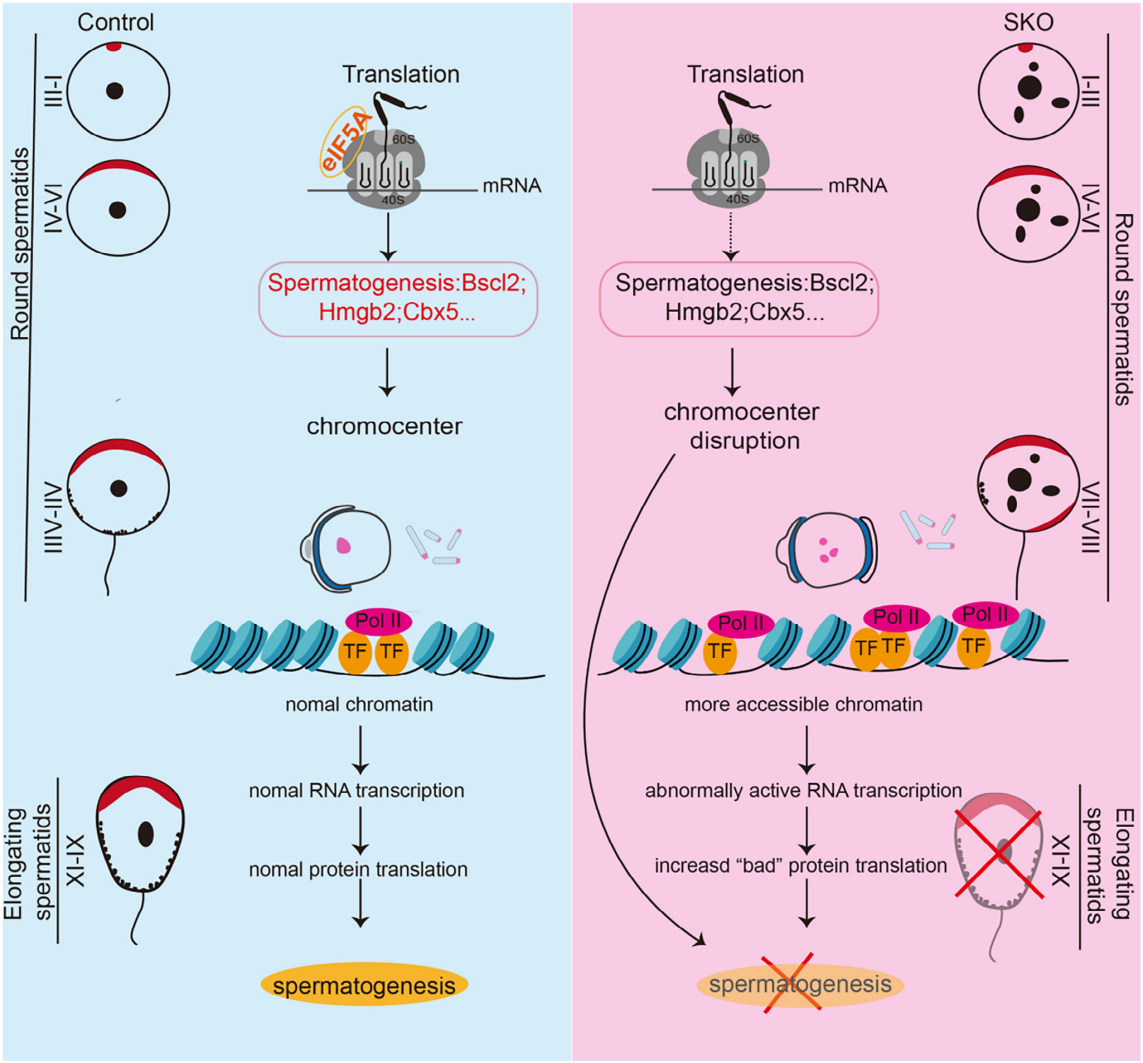
Model of proposed eIF5A function in mouse spermatogenesis. EIF5A is essential for the translation of heterochromatin‐ and acrosome‐associated proteins during spermatogenesis. Loss of eIF5A leads to disruption of acrosomal structure and single chromocenter formation, increased chromatin accessibility, transcriptional dysregulation, and elevated expression of aberrant proteins, ultimately resulting in failed spermatogenesis at the round spermatid stage.

Spermatogenesis demands exquisitely coordinated gene regulation at both transcriptional and translational levels to drive the dramatic morphological transformations of spermatids. Our findings reveal an intricate interplay between these regulatory layers during spermiogenesis, with eIF5A emerging as a critical molecular mediator bridging transcription and translation. In this study, we establish eIF5A as an indispensable regulator of this process, essential for translation elongation and, crucially, for facilitating chromocenter formation. Conditional *Eif5a* knockout causes specific arrest at the round spermatid stage, concomitant with aberrant acrosome and manchette assembly, widespread increases in chromatin accessibility, and dysregulation of key genes at mRNA and protein levels (**Figure** **7**). Our proteomic data directly demonstrate that eIF5a loss leads to a drastic failure in synthesizing key chromatin factors (e.g., Bscl2, Hmgb2). This primary translational impairment initiates chromocenter fragmentation, which is mechanistically supported by our ATAC‐seq results showing consequent genome‐wide increases in chromatin accessibility (Figure 6). Thus, the experimental evidence establishes a clear causal hierarchy beginning with translational arrest of specific chromatin proteins, leading to chromocenter disintegration, subsequently increasing chromatin accessibility, and thereby resulting in transcriptional dysregulation that ultimately manifests as the observed acrosomal and manchette defects. The unimpaired meiotic progression in SKO mice suggests potential functional compensation by other translation factors during meiosis, underscoring the stage‐specific complexity of translational regulation in spermatogenesis.

While prior work in yeast and somatic cells defined eIF5A's foundational roles in translation elongation and termination,^[^ ^16^, ^44^ ^]^ our results unveil a novel and essential function: eIF5A acts as a central orchestrator of chromocenter‐directed nuclear remodeling during terminal spermatid differentiation. Post‐meiotic nuclear compaction, critical for sperm function, is tightly coupled with extensive chromatin reorganization,^[^ ^20^, ^21^ ^]^ including chromocenter formation where centromeric heterochromatin aggregates into one or two chromocenters.^[^ ^20^, ^23^, ^45^ ^]^ Here, we provide the first direct evidence that eIF5A is essential for proper chromocenter formation. Its ablation results in fragmented chromocenters. Mechanistically, this defect aligns with impaired translation of key chromocenter regulators (BSCL2 and HmgB2), whose deficiencies phenocopy the fragmented chromocenters seen in SKO mice.^[^ ^25^, ^40^ ^]^ Notably, BSCL2 deficiency also causes acrosomal defects. Furthermore, our ATAC‐seq analysis reveals that chromocenter disruption in SKO round spermatids correlates with global increases in chromatin accessibility and substantial transcriptional dysregulation, highlighting the far‐reaching consequences of this initial nuclear defect.

This observed transcriptional landscape presents a paradox: despite widespread chromatin relaxation and the predominance of upregulated transcripts in SKO testes, a significant cohort of genes (n = 4584) were downregulated (Figure 6A). Several mechanisms likely contribute. First, the selective reduction in chromatin accessibility at specific loci could directly repress transcription. Second, our proteomics uncovered broad downregulation of transcription factors, potentially impairing transcriptional activation programs. Third, eIF5A's established roles extend beyond translation to mRNA stability and decay.^[^ ^46^, ^47^ ^]^ Impaired translational elongation due to eIF5A deficiency might accelerate the degradation of inefficiently translated mRNAs, potentially via pathways like nonsense‐mediated decay (NMD),^[^ ^39^ ^]^ thereby reducing transcript abundance independently of chromatin state.

Our proteomic data also indicates that eIF5A deficiency perturbs the expression of mitochondrial‐associated proteins (Figure 4C), suggesting an additional regulatory layer for germ cell development that merits future investigation. Intriguingly, spermidine, found at exceptionally high levels in the testis, is critical for spermatogenesis and sperm motility.^[^ ^48^, ^49^, ^50^ ^]^ Crucially, eIF5A is the sole known cellular target of hypusination, a unique post‐translational modification absolutely dependent on spermidine.^[^ ^48^, ^51^ ^]^ This strong evidence suggests that spermidine may exert its key reproductive effects, at least in part, through eIF5A hypusination. Moreover, the interplay between spermidine metabolism and eIF5A hypusination likely forms a critical regulatory node, integrating cellular energy status, redox balance, and protein remodeling to orchestrate germ cell maturation.^[^ ^52^, ^53^ ^]^ Future studies should prioritize: 1) determining if hypusination modulates eIF5A's binding affinity for specific mRNA targets or protein partners critical for spermatogenesis, and 2) mapping the temporal and spatial dynamics of eIF5A hypusination throughout spermatogenesis to elucidate its stage‐specific functions. Understanding these mechanisms may not only illuminate fundamental aspects of male reproductive biology but also illuminate potential therapeutic avenues for infertility or spermatogenic disorders linked to dysregulated spermidine metabolism or eIF5A dysfunction.^[^ ^33^, ^54^ ^]^

Recent studies have reported that pathogenic variants in EIF5A and its hypusination enzymes (DHPS and DOHH) cause neurodevelopmental disorders characterized by microcephaly, epilepsy, and developmental delay.^[^ ^55^, ^56^ ^]^ Impaired eIF5A function disrupts the translation of poly‐proline‐rich proteins and can be partially rescued by spermidine supplementation.^[^ ^57^ ^]^ In addition, analysis of the gnomAD database identified 158 EIF5A variants, including predicted loss‐of‐function and missense/inframe indels, suggesting potential clinical heterogeneity. Together, these findings imply that dysregulation of the eIF5A–spermine/hypusination pathway may also contribute to human male infertility and warrants further investigation in genetic and clinical cohorts.

## 4 Experimental Section

### Mice Generation and Maintenance

*Eif5a^flox/+^* mice on a C57/6J background were generated in Cyagen Biosciences using a CRISPR‐Cas9‐mediated genome editing system and *Stra8‐GFPCre* mice were a generous gift from Professor Minghan Tong of the Chinese Academy of Sciences. *Eif5a^fl/−^* mice were produced by *Eif5a^flox/flox^* mice crossing with Stra8‐*GFPCre* knockin mice, The *Stra8‐GFPCre* mice were used to generate *Eif5a* germ cells deficiency mice (*Eif5a* SKO mice) with a deletion of 3069 base pairs (6350‐7334), which specifically targeted the exons 2 to 7 of the *Eif5a* gene (NM_0 011 66589). The founders were tested with genotyping performed by polymerase chain reaction (PCR) and followed by DNA sequence analysis. All mice were housed in the Model Animal Research Center of Shandong University and exposed to a 12‐hour light‐dark cycle under specific pathogen‐free (SPF) conditions, with controlled temperature (22 ± 1 °C) and humidity (40–70%). All experimental protocols were approved by the Regional Ethics Committee of Shandong University.

### Fertility Assessment

Fertility was assessed in control and SKO mice starting at 8 weeks of age. Each SKO male was housed with two 8–12week‐old WT C57BL/6J female mice. Female mice were monitored daily for the presence of vaginal plugs, and those with plugs were placed in individual cages for pregnancy tracking. The fertility test was conducted for a minimum of 6 months.

### Tissue Collection and Histological Analysis

After euthanasia, mice testes and epididymides were dissected and fixed in 4% paraformaldehyde (p1110, Solarbio, Beijing, China) for 24 hours. Tissues were then dehydrated, paraffin‐embedded, and sectioned into 5 µm slices before being mounted onto deparaffinized glass slides. The slides were dried at 65 °C for 30 minutes before further processing. The tissue sections on slides were deparaffinized and rehydrated using xylene and a gradient of ethanol (100%, 95%, 90%, 80%, 70%). Periodic acid‐Shiff (PAS) and /or hematoxylin staining were followed by histological analysis.

### TUNEL Assay

TUNEL staining was performed following the manufacturer's instructions (keyGEN BioTECH, #KGA7072).

### Immunofluorescence Staining and Antibodies

Testicle sections were fixed in 4% paraformaldehyde, permeabilized with 0.1% Triton X‐100, incubated with 5% BSA in PBS buffer, followed by primary antibody incubation at 4 °C for 12 hours. Primary antibodies used for immunofluorescence were as follows: EIF5A (dilution 1:500, Proteintech, #11309‐1‐AP), MVH (dilution 1:500, Abcam, #ab13840) SYCP3(dilution 1:500, Abcam, #ab97672, #ab15093), SYCP1(dilution 1:500, Abcam, #ab15090) γH2AX(Ser139) (dilution 1:2000, Millipore, #05‐636)

The primary antibodies were detected using secondary antibodies conjugated with Alexa Fluor 488 or 594 (dilution 1:500, Abcam #ab150084, #ab150077, #ab150113, and #ab150120) for 1 hour at room temperature. Subsequently, the slides underwent several washes with PBS and were mounted using a mounting medium with DAPI‐aqueous, fluoroshield (Abcam, ab104139). Immunofluorescence images were captured immediately using either an LSM 780/710 microscope (Zeiss) or an SP8 microscope (Leica).

### Western Blotting

Tissues were collected to prepare protein extracts from C57BL/6 mice and lysed in NP‐40 lysis buffer (Beyotime, P0013F) plus protease inhibitors (Roche, 0 469 313 2001). The tissue was homogenized and then kept on ice for 30 minutes, followed by centrifugation at 12,000 rpm for 20 minutes at 4 °C. Then transfer supernatant to a new tube for subsequent immunoblotting. Equal amounts of proteins were electrophoresed on 10% SDS‐polyacrylamide gels and then transferred to polyvinylidene fluoride membranes (Millipore, USA). The membranes were blocked with 5% nonfat milk at room temperature for an hour and then incubated overnight at 4 °C with the primary antibodies. Following three washes with TBST, they were further incubated with secondary antibodies at room temperature for one hour. Immunoreactive bands were detected and analyzed using a Bio‐Rad ChemiDoc MP imaging system and Image Lab software (Bio‐Rad, USA). The primary antibodies and for immunoblotting included:EIF5A (1:1000 dilution, Proteintech, #11309‐1‐AP), RPL10A (1:1000 dilution, Abclonal, #A20944), BSCL2 (1:1000 dilution;Abclonal, #A14583), HMGB2 (1:1000 dilution, Proteintech, #14597‐1‐AP), CBX5 (1:1000 dilution, Proteintech, #11831‐1‐AP), β‐ACTIN (1:50 000 dilution, Proteintech, #881115‐1‐RR), SPATA1(1:1000 dilution, Proteintech, #24980‐1‐AP), SPACA3(1:1000 dilution, Proteintech, #21137‐1‐AP), SPACA9(1:1000 dilution, Proteintech, #26034‐1‐AP). The secondary antibodies were: HRP‐conjugated goat anti‐ Mouse IgG(1:10 000 dilution, Beyotime, #A0216) and HRP‐conjugated goat anti‐ rabbit IgG (1:10 000 dilution, Beyotime, #A0258).

### Electron Microscopy

The testicular samples for transmission electron microscopy (TEM) analysis were initially fixed in a solution containing 2.5% phosphate‐buffered glutaraldehyde. Then the samples underwent a sequential dehydration process using an ethanol gradient of increasing concentrations (50%, 70%, 90%, and finally 100%). Subsequently, the dehydrated samples were infiltrated with 100% acetone. After embedding in Epon 812, ultrathin sections were cut using an ultramicrotome, followed by staining with uranyl acetate and lead citrate. Finally, the prepared samples were examined and imaged using a transmission electron microscope (TECNAI‐10, Philips) operating at an accelerating voltage of 80 kV.

### Fluorescence‐Activated Cell Sorting (FACS) of Round Spermatids

The purification of round spermatids from 28‐day‐old control and *Eif5a* SKO mice was conducted using Fluorescence‐activated cell sorting (FACS), as previously described.^[^ ^58^, ^59^, ^60^ ^]^ After removing the tunica albuginea, the testis was placed in 5 mL DPBS containing 120 U mL^−1^ type I collagenase and mixed at 35 °C for 10 minutes. Digestion of the testis was then carried out using 5 mL 0.25% trypsin plus 0.1 mL DNase I (5 mg mL^−1^) at 35 °C for 10 minutes, followed by the addition of 0.5 mL fetal bovine serum (FBS) to stop the reaction. The suspension was filtered through a 40 µm cell strainer. The cell suspension was centrifuged at 4 °C for 5 minutes at 500 ×g, and the supernatant was removed. The cell pellet was resuspended in 1 mL DMEM, and then the cell concentration was determined using Countess 3 Automated Cell Counter (Thermofisher, A49891). Then approximately 20 mL of DMEM containing Hoechst 33 342 (3 µg/10^6 cells) was added. The mixture was rotated at 35 °C for 20 minutes, followed by centrifugation at 4 °C for 5 minutes at 500 ×g. Cells were resuspended in PBS at a final concentration of 10^5 cells mL^−1^ for sorting. Fluorescence‐activated cell sorting (FACS) was performed by BD‐FACSAria Fusion, and the fluorescently labeled cell population was collected into 1.5 mL LoBind microcentrifuge tubes containing 0.5 mL PBS. The gating strategy applied was as follows16: cells were initially gated based on forward scatter (FSC) and side scatter (SSC) to exclude debris and select the main population of singlet cells. Doublets were subsequently excluded by analyzing FSC‐Width versus FSC‐Height. Finally, round spermatids were haploid (1C) cells. Staining with Hoechst 33 342 allows for the clear discrimination of the haploid cell population (round spermatids) from the diploid (2C, e.g., somatic cells, spermatogonia) and tetraploid (4C, e.g., primary spermatocytes) populations based on fluorescence intensity. The cell suspension was centrifuged, removing some supernatant, leaving approximately 10 µL of PBS.

### Sucrose Gradient Analysis

The sucrose gradient analysis of testicular extract was performed as described.^[^ ^4^ ^]^ Briefly, mouse testicular tissue was homogenized in extraction buffer containing 100 mM NaCl, 50 mM Tris‐HCl (pH 7.5), 5 mM MgCl_2_, 1% Triton X‐100, and 100 µg ml^−1^ cycloheximide. The homogenate was then centrifuged at 13000 ×g for 2 minutes to remove nuclear debris. The resulting extract was layered onto a 10% to 60% sucrose gradient and centrifuged at 38,000 rpm in an SW41 rotor for 2 hours at 4 °C. For puromycin treatment, tissues were homogenized in extraction buffer and then incubated with 500 µM puromycin at 30 °C for 30 minutes. Twelve fractions were collected using a piston gradient fractionator (Biocomp, Fredericton, Canada), and each fraction was analyzed for specific proteins and RNA by protein blotting and RT‐qPCR, respectively.

### Sample Preparation and Enzymatic Digestion for Protemics

Round spermatids were isolated from 28‐day‐old C57BL/6 male mice by fluorescence‐activated cell sorting (FACS) and subjected to proteomic analysis, with all experimental and analytical procedures performed by Luming Biotech. The isolated round spermatids were lyzed in BT lysis buffer containing PMSF, followed by sonication. After protein reduction with Dithiothreitol (DTT) (192.5 mg of DTT dissolved in 50 mL deionized water, aliquoted into 200 µL tubes, and stored at ‐20 °C to avoid repeated freeze‐thaw cycles) at 55 °C for 30 minutes, samples were alkylated using Iodoacetamide (IAA) (369.9 mg of Iodoacetamide dissolved in 10 mL deionized water, aliquoted into 200 µL tubes, and stored at ‐20 °C in the dark) at room temperature in the dark for 30 minutes. Protein digestion was performed by adding trypsin and incubating at 37 °C with shaking at 1500 rpm. The digested peptides were desalted and prepared for mass spectrometry analysis.

### Mass Spectrometry Measurement

The timsTOF Pro2 was used for data acquisition in positive ion mode with parallel accumulation‐serial fragmentation (PASEF) DIA mode. The capillary voltage was set to 1400 V, with the MS1 and MS2 scan range spanning 100–1700 m/z. The ion mobility window range (1/K0) was set from 0.7 to 1.4 Vs/cm^2^. Ion accumulation and release time were set to 100 ms to achieve nearly 100% ion utilization efficiency. A full PASEF cycle included 10 PASEF MS2 scans, with a total cycle time of 1.1 s. In the method setup, polygonal frames were applied to efficiently filter low‐quality charge‐to‐mass ratios (m/z) and singly charged ions. During precursor ion selection, the target intensity for parent ions was set at 20000, with an intensity threshold of 2500 for selection into MS2 analysis. In each 100 ms PASEF cycle, 4 quadrupole isolation windows and 4 tims drift time windows were set, with a quadrupole isolation window width of 28 Da. The collision energy was linearly adjusted based on ion mobility (1/K0), ranging from 20 to 59 eV for ions with drift times (1/K0) from 0.6 to 1.6 Vs/cm^2^.

### Data Analysis for Protemics

DIA raw data were processed using Spectronaut Pulsar 18.4 software (Biognosys). The sequence database used for analysis was uniprot‐Mus musculus‐10090‐2024.2.1.fasta. DirectDIA mode was applied without database construction for data analysis. Detailed procedures for data processing can be found in the software user manual.

### Functional Enrichment Analysis

For each given list of genes, functional enrichment analysis of differentially expressed genes (DEGs) were was performed and differentially translated efficiency genes (DTEGs) using the online tool Metascape (http://metascape.org)^[^ ^61^ ^]^ with the following ontology sources: GO Biological Processes, GO Cellular Components, GO Molecular Function. All genes in the genome were used as the enrichment background. Terms with a p value < 0.01, a minimum count of 3, and an enrichment factor > 1.5 (the enrichment factor was the ratio between the observed counts and the counts expected by chance) were collected and grouped into clusters based on their membership similarities. Hypergeometric tests and Benjamini Hochberg‐based false discovery rate (FDR) control procedures were used to determine the enrichment of each term.

### RNA Extraction, RT‐qPCR

Total RNA was isolated from whole testes or sorted cells using the FastPure Cell/Tissue Total RNA Isolation Kit V2 (Vazyme, RC112‐01), following the manufacturer's protocol.

For RT‐qPCR, total RNA was reverse‐transcribed into cDNA using the HiScript III RT SuperMix for qPCR (+gDNA wiper) (Vazyme, R323‐01). It was carried out using the SYBR Green Premix Pro Taq HS qPCR Kit (AG, 11 701) on the LightCycler@96 Real‐Time PCR system (Roche), as per the manufacturer's guidelines. Actin (LOC107788267) served as the internal control, and each experiment was performed in triplicate for technical consistency. Relative gene expression was calculated using. The primers were provided in Supporting Information.

### Targeted Profiling of RNA translation (TPRT)

The targeted profiling of RNA translation (TPRT) assay was conducted as previously described.^[^ ^42^, ^62^ ^]^ control and SKO round spermatids, sorted by FCAS, were washed with ice‐cold PBS containing 100 µg mL^−1^ cycloheximide. The cells were then lysed in polysome buffer with 1 mg mL^−1^ cycloheximide, snap‐frozen in liquid nitrogen, thawed on ice, and collected. After clarification by centrifugation, the samples were diluted to 500 ng in 100 µL lysis buffer. RNase I (Invitrogen, 1000 U) and TurboDNase (Invitrogen, 10 U) were added, and the samples were incubated for 1 hour at 4 °C. Ribosomal footprints were extracted using TRIzol reagent (Invitrogen) and purified through isopropanol precipitation. Reverse transcription was performed with footprint‐specific primers targeting the region around the translation initiation site, as illustrated in Supporting Information Appendix, using SuperScript III reverse transcriptase (Invitrogen) with a ramped temperature protocol from 40 °C to 50 °C for 1 hour. The RNA template was hydrolyzed with NaOH, and cDNA was purified by isopropanol precipitation. qPCR was conducted using a ribosome footprint‐specific forward primer targeting the translation initiation site and a common reverse primer, as shown in Supporting Information Appendix, with the SYBR‐Select master mix (Applied Biosystems).

### Smart‐seq2

For Smart‐seq2, cell lysis, mRNA reverse transcription, and cDNA amplification were performed using the Discover‐sc WTA Kit V2 (Vazyme, N711). Following this, cDNA fragmentation and adapter ligation were carried out with the TruePrep Flexible DNA Library Prep Kit for Illumina (Vazyme, TD504). All procedures were conducted in strict accordance with the manufacturer's protocols. The Smart‐seq2 library sequencing was performed by Novogene on Illumina platforms, generating 150 bp paired‐end reads.

### Smart‐seq2 Data Analysis

Raw data (raw reads) of fastq format were first quality control analyzed using FASTQC (http://www.bioinformatics.babraham.ac.uk/projects/fastqc/). Raw sequencing reads were trimmed to remove low‐quality bases by Trim Galore (version 0.6.4)(https://github.com/FelixKrueger/TrimGalore) in PAIRED‐end mode. All the downstream analyses were based on clean data with high quality. Paired‐end clean reads were aligned to the reference genome (GRCm38) by HISAT2 (version 2.0.5).^[^ ^63^ ^]^ FeatureCounts (version 1.5.0‐p3)^[^ ^64^ ^]^ of the subread package was used to count the reads numbers mapped to each gene. And then FPKM of each gene was calculated by R script based on the length of genes and reads count.

### Ribo‐seq Experiment, Library Preparation, and Sequencing

The Ribo‐seq was performed as previously described.^[^ ^65^ ^]^ Round sperm samples were lysed in ice‐cold lysis buffer (20 mM Tris‐HCl pH 7.4, 150 mM NaCl, 5 mM MgCl_2_, 1 mM DTT, 100 µg ml^−1^ CHX, 1% Triton X‐100, 25 U ml^−1^ Turbo DNase) for 4 hours on ice. Lysates were clarified at 20000 ×g for 10 minutes at 4 °C, treated with RNase I (1 µl of 100 U/µl) at room temperature for 45 minutes, and the reaction terminated with 10 µl SUPERase•In. Samples were layered onto a 700 µl sucrose cushion (1 M sucrose, 20 mM Tris‐HCl pH 7.4, 150 mM NaCl, 5 mM MgCl_2_, 1 mM DTT, 100 µg ml^−1^ CHX, 20 U ml^−1^ SUPERase•In) and centrifuged at 260000 ×g, 4 °C for 4 hours. Pellets were resuspended in 50 µl precipitate buffer, and RPFs were extracted using TRIzol and chloroform. RPFs were precipitated with isopropanol and glycogen at ‐20 °C and resuspended in nuclease‐free water.

RPFs were separated on a 15% TBE‐urea polyacrylamide gel (200 V, 65 min), and RNA was extracted from gel slices using RNA extraction buffer, precipitated, and resuspended in nuclease‐free water. Sequencing libraries were prepared using the CATS Small RNA‐seq Kit or D‐Plex Small RNA‐seq Kit, followed by purification with AMPure XP. Barcoded libraries were pooled and sequenced on Illumina platforms, generating 150 bp paired‐end reads.

### Ribo‐seq Data Processing

Raw reads were trimmed with cutadapt v4.5^[^ ^66^ ^]^ with parameters: cutadapt ‐u 16 ‐n 3 ‐a AGATCGGAAGAGCACACGTCTG ‐a AAAAAAAA ‐o <output.file> <input.file>, and the trimmed reads were sequentially mapped to mouse rRNA sequences (mm10) using Bowtie2 v2.2.5^[^ ^67^ ^]^ with parameters –seedlen = 23. Those aligned to rRNA were discarded, and the remaining reads were mapped to transcriptome of mm10 using STAR v2.7.10b^[^ ^68^ ^]^ with parameters –outFilterMismatchNmax 2 –outFilterMultimapNmax 20 –outFilterMatchNmin 16 –alignEndsType EndToEnd. The gene expression levels were then calculated by cufflinks v2.2.1^[^ ^69^ ^]^ based on the annotation of CDS region, defined by mm10 refFlat database from the UCSC genome browser. Average FPKM for replicates were next calculated and as long as expression was detected in one duplicate sample, it was regarded as genes detected by Ribo‐lite.

Spearman correlations were calculated between duplicate samples and different samples. 3‐nt periodicity was assessed and the offset was determined. The 3‐nt periodicity of RPF reads based on their P‐site alignment was used as strong evidence for activated translation. The 3‐nt periodicity of all Ribo‐seq samples were evaluated using the RiboCode v1.2.15 program.^[^ ^70^ ^]^ RiboCode v1.2.15 also calculated the percentage of periodic reads and identified all translated ORFs, including new, unannotated ORFs such as uORFs.

### Metagene Analyses of Ribosome Profiling Data

Metagene analyses of ribosome profiling data were performed by MetageneAnalysis of RiboMiner v0.2.3.2^[^ ^71^ ^]^ with “‐u 0 ‐d 500 ‐l 100 ‐n 10 ‐m 1” parameters. Specifically, for genes that exhibited more than one transcript, only the longest transcript was used in all analyses. Only the transcripts with length over 100 codons and RPKM more than 10 in the whole CDS region were considered. The ribosome densities were normalized to reads per million mapped reads (RPM).

### Codon Optimality Analysis

To observe whether there were differences between different codons or amino acids (AA), the ribosome density was calculated at each codon or AA by RiboDensityAtEachKindAAOrCodon of RiboMiner v0.2.3.2.^[^ ^71^ ^]^ In order to select those triplete amino acid (tri‐AA) motifs that were rich in ribosomes, the ProcessPausingScore function was used to complete this work and output the position weight matrix (PWM) of the E, P, and A sites of triplete amino acid (tri‐AA) motif, and then used Seq2Logo^[^ ^72^ ^]^ to perform motif Logo drawing.

### Polarity Calculation

To estimate ribosome tracking the mean score was calculated by RiboMiner. The PolarityCalculation of RiboMiner v0.2.3.2 was used^[^ ^71^ ^]^ to evaluate the ribosomes of all genes for each reconstructed version and analyzed the changes in scores after knocking out *Eif5a* by comparing the scores in the SKO group and scores. Here p value was calculated by t test from the mean of repeated samples.

### Assay for Transposase‐Accessible Chromatin Using Sequencing (ATAC‐seq) Analysis

Round spermatids were sorted by FCAS from three mice in each of the control and SKO groups. ATAC‐seq was performed following the manufacturer's instructions (Vazyme, TD711). The library preparations were sequenced on an Illumina HiSeq platform and 150 bp paired‐end reads were generated.

Adaptor sequences were removed from the reads using Trim Galore (https://github.com/FelixKrueger/TrimGalore/releases). The trimmed reads were then aligned to the reference genome with HISAT2 (http://github.com/infphilo/hisat2).^[^ ^73^ ^]^ with standard parameters. SAMtools 1.16^[^ ^74^ ^]^ was used to convert the comparison files into bam format, and Picard (http://broadinstitute.github.io/picard/) was used to delete the duplicates generated by PCR of the above file to obtain clean reads. All peak calling was performed using model‐based analysis of chip‐seq (MACS2).^[^ ^75^ ^]^ To identify enriched regions from biologically replicated samples in SKO ATAC‐seq, the Irreproducible Discovery Rate (IDR) method was used (https://github.com/nboley/idr),^[^ ^76^ ^]^ where the parameter setting of MACS2 should not be too strict to identify more peaks. Following this method, peak calling was performed using MACS2 with relaxed conditions (–shift −100 –extsize 200 –nomodel ‐B –SPMR ‐g mm) on each of the two replicates of the pooled dataset. IDR analysis was then performed, and reproducibility was checked. To obtain the final peak sets, the data were sorted by ‐log10 (p value). The threshold recommended by the authors was used. The peaks were annotated and visualized using the R package ChIPseeker.^[^ ^77^ ^]^ Differential ATAC‐seq peak analysis was performed using the (version 3.14.0).^[^ ^78^ ^]^ Peaks were normalized by library size using the DBA_NORM_LIB method to correct for technical variation across samples, and differential binding regions were identified with FDR ≤ 0.05 and |log_2_fold change| ≥ 1. Motif calling was performed using the Homer command annotate Peaks and Homer command find Motifs Genome (https://github.com/nboley/idr) with parameter “‐size 200 and ‐len 8, 10, and 12”.^[^ ^79^ ^]^

### H3K4me3 CUT&Tag

The CUT&Tag assay for H3K4me3 was performed using Hyperactive Universal CUT&Tag Assay Kit (Vazyme, TD904) with minor modifications. Briefly, round sperm samples in testes of wild‐type and *Eif5a* SKO mice were collected using flow sorting. Approximately 50000 cells per reaction were resuspended in wash buffer, after incubating the cell suspension with ConA beads for 8 minutes, the magnetic beads were enriched and then coated with an H3K4me3 antibody (Cell Signaling Technology, 9751S, 1:50 dilution). The coated magnetic beads were incubated at 4 °C overnight. The next day, after incubating with the secondary antibody and pA/G‐Tnp Pro successively for 1 hour, the fragmentation reaction was carried out. DNA was purified using DNA Extract Beads Pro, the purified DNA was then used as a template for PCR amplification with indexed primers to generate the sequencing library (Vazyme, TD202). The final PCR‐amplified libraries were purified using VAHTS DNA Clean Beads (Vazyme, N411), quantified, and assessed for quality. Libraries were sequenced on an Illumina platform to obtain paired‐end reads.

### H3K4me3 CUT&Tag Data Processing and Analysis

Sequencing reads were quality‐checked using FastQC. Clean reads were aligned to the mm10 mouse genome using Bowtie2,^[^ ^80^ ^]^ followed by file conversion and sorting with samtools. Normalized coverage tracks were generated using deepTools 3.5.5,^[^ ^81^ ^]^ and peaks were called with MACS3 3.0.2.^[^ ^82^ ^]^ Peak annotation and visualization were performed using ChIPseeker.^[^ ^83^ ^]^ Differential peak analysis between WT and KO groups was conducted using DiffBind^[^ ^78^ ^]^ based on edgeR frameworks.

To assess cross‐omics concordance, differential peaks identified from CUT&Tag (H3K4me3) and ATAC‐seq were annotated to their nearest genes and intersected using bedtools. The overlap ratio between the two datasets was visualized as a Venn diagram. Log_2_(fold‐change) values derived from DiffBind analyses were compared between CUT&Tag and ATAC‐seq, and visualized using ggplot2^[^ ^84^ ^]^ in four‐quadrant correlation plots. Correlation coefficients (Pearson's r) were computed to quantify the degree of concordance between histone modification and chromatin accessibility changes.

### Single‐Cell RNA‐Seq Analysis

The raw UMI count matrix of the human testis dataset^[^ ^85^ ^]^(GSE112013) and the raw count matrix of the mouse testis dataset^[^ ^32^ ^]^ (GSE107644) were analyzed using Seurat (v4.4.0)^[^ ^86^ ^]^ in R. Standard preprocessing included normalization (LogNormalize, scale.factor = 10000), identification of highly variable genes (vst, n = 2000), data scaling (ScaleData), principal component analysis (RunPCA), graph‐based clustering (FindNeighbors, FindClusters), and UMAP visualization (RunUMAP). Cell types in the human dataset were annotated according to canonical markers described in the original publication, while the cell‐type annotations in the mouse dataset were directly adopted from the original study. The VlnPlot function was used to visualize EIF5A/Eif5a expression across cell types.

### Phylogenetic Analysis

Protein sequences of EIF5A from multiple species were obtained from the NCBI database in FASTA format. Multiple sequence alignment was performed using ClustalW implemented in MEGA11.^[^ ^87^ ^]^ The best substitution model was determined by the Find Best Protein Models (ML) function based on the Bayesian Information Criterion (BIC), and the JTT model was selected. The Neighbor‐Joining (NJ) method^[^ ^88^ ^]^ was used to construct the phylogenetic tree with 1000 bootstrap replicates.^[^ ^89^ ^]^ The bootstrap consensus tree was used to illustrate the evolutionary relationships among species.

### Statistical Evaluation

Quantitative data were obtained from at least three independent biological replicates (n ≥ 3), with the specific sample size for each experiment detailed in the corresponding figure legend. All quantitative data were expressed as the mean ± Standard Deviation (SD). All statistical analyses were conducted using GraphPad Prism software (version 8.0, GraphPad, San Diego, CA, USA). Differences between the two groups were assessed using a two‐tailed, unpaired Student's t‐test. The threshold for statistical significance was set at P < 0.05.

## Conflict of Interest

The authors declare no conflict of interest.

## Author Contributions

Y.C., T.L., and Q.F. contributed equally to this work. Y.L.C. was involved in conceptualization, the majority of experimentation, data collection, analysis, and writing—original draft, review & editing. T.T.L. analyzed Ribo‐seq, Smart‐seq2, and ATAC‐seq data. Q.F. participated in writing the manuscript, revising its intellectual content, and conducting a portion of the data analysis. Z.Y.B. performed ATAC‐seq experiments. F.K. performed Ribo‐seq experiments. And M.Y.Z., H.Z.L., and W.W. performed part of the genotyping. H.T.Q., Y.X.G., W.B.L., and X.F.C. participated in manuscript revision and responding to reviewers' comments. H.B.L., X.W., X.H.J., and Z.J.C. were responsible for supervision, project administration, resources, and funding acquisition. All authors have read and approved the final version of the manuscript.

## Supporting information



Supporting Information

## Data Availability

The data that support the findings of this study are available from the corresponding author upon reasonable request.
