# Supplementary material for: EIF5A Couples Translational Control With Transcriptional Reprogramming Through Chromocenter Reorganization During Spermiogenesis
Source: Adv Sci (Weinh). 2026 Jan 4;13(9):e17423. doi: 10.1002/advs.202517423 (PMC12903998; doi:10.1002/advs.202517423)
Supplement: Supplementary file 1 — Supporting Information [file ADVS-13-e17423-s001.docx]

**Supplementary information**

**
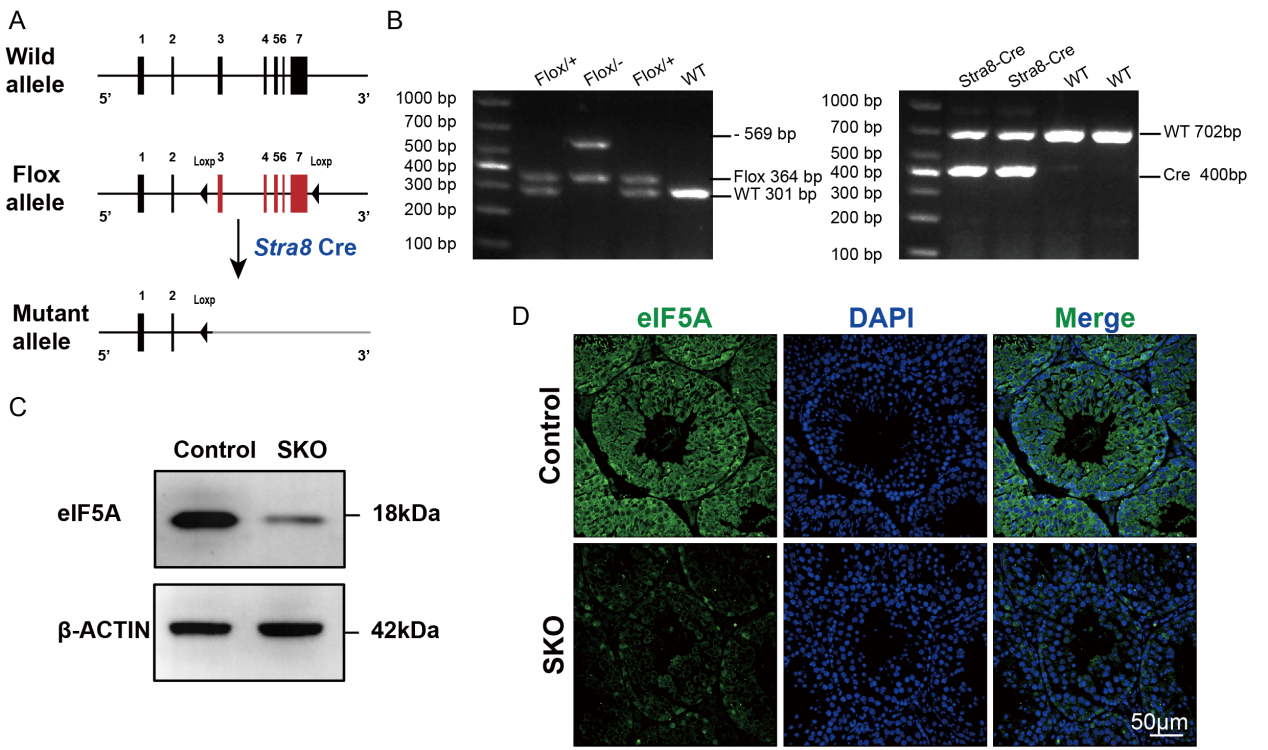
Figure S1 Generation of germ cell-specific *Eif5a* knockout mouse model.**

1. Hybrid approach used to generate the *Eif5a* SKO mice with *Eif5a* specifically deleted in germ cells.
2. Genotype identification of *Eif5a* conditional knockout mice using the *Eif5a*-Flox and *Stra8-GFPCre* primers. The primer sequences are listed in Supplementary Table S3.
3. Western blotting against eIF5A in testes from 12-week-old control and *Eif5a* SKO testes. β-Actin served as the loading control.
4. EIF5A immunofluorescence in control and *Eif5a* SKO adult testis. Scale bar, 50 μm.

**
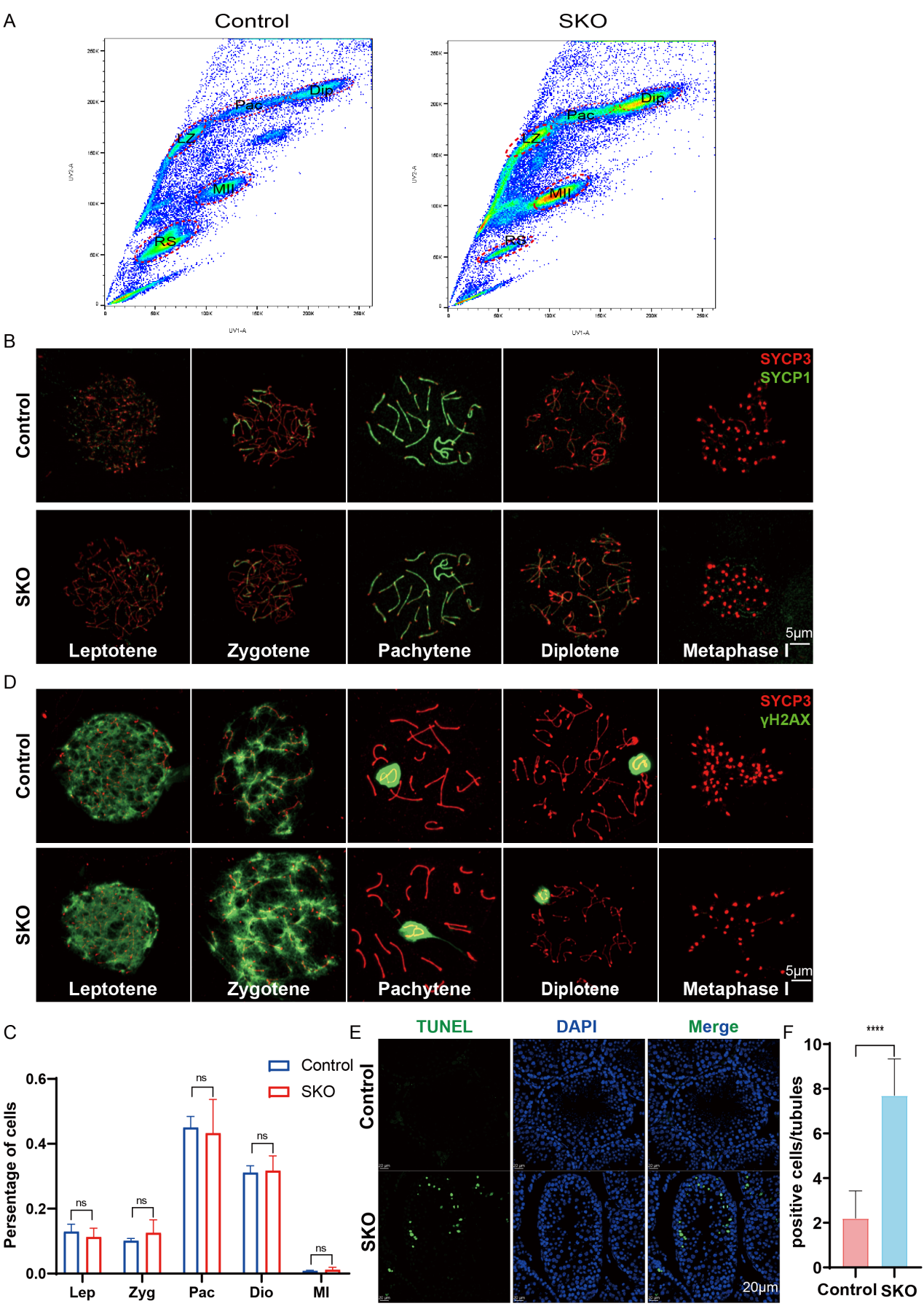
**

**Figure S2 Conditional knockout of *Eif5a* in testes did not affect meiosis progression.**

1. A representative Control (left) and SKO (right) Hoechst 33342 FACS profile is shown. The various meiotic cell populations that can be purified using this method for further study are indicated:leptotene-zygotene (L/Z), Pachytene(Pac) ,diplotene(Dip), round spermatids (RS) and Metaphase II(MII).
2. Chromosome spreads of control and *Eif5a* SKO spermatocytes at P20 were stained for SYCP3 and SYCP1. Scale bars, 5 μm.
3. Percentages of spermatocytes at the leptotene (Lep), zygotene (Zyg), pachytene (Pac), diplotene (Dip) and Metaphase I(MI) stages from control and *Eif5a* SKO mice at P20. Data are presented as mean ± SD. *n* = 3 biological replicates. P value was calculated by a two-tailed, unpaired Student's *t*-test , ns, not significant.
4. Co-immunofluorescent staining of SYCP3 with γH2AX in spermatocyte chromosome spreads from control and *Eif5a* SKO mice at P20. Scale bars, 5 μm.
5. TUNEL assays of testis sections from 12-week-old control and *Eif5a* SKO mice. Scale bar, 20 μm.
6. Quantification of the number of TUNEL-positive cells per tubule. Data are presented as the mean ± SEM (n = 3 biological replicates). ****P < 0.0001 by two-tailed Student’s *t*-test.

**
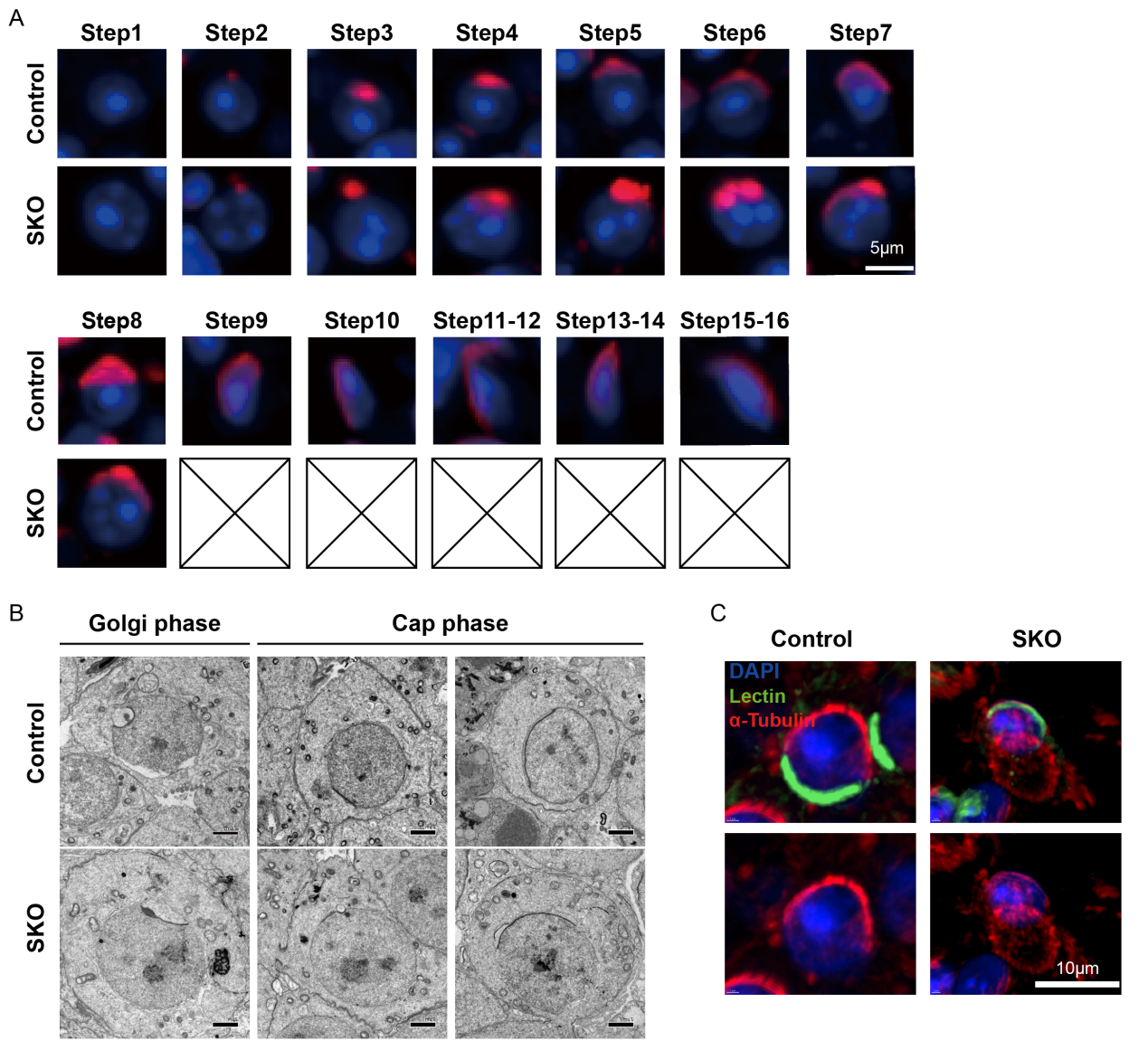
**

**Figure S3 *Eif5a* deficiency impairs acrosome formation, manchette development, and spermatid elongation.**

1. The absence of *Eif5a* resulted in multiple defects during acrosome and nuclear development as observed in testis sections stained with PNA and DAPI. Scale bars, 5 µm .
2. Transmission electron microscopy showing the ultrastructure of the acrosome in spermatids from control and *Eif5a* SKO males.

Assembly and morphology of the manchette in spermatids highlighted by decoration of α-tubulin, Scale bar, 5 μm.

**
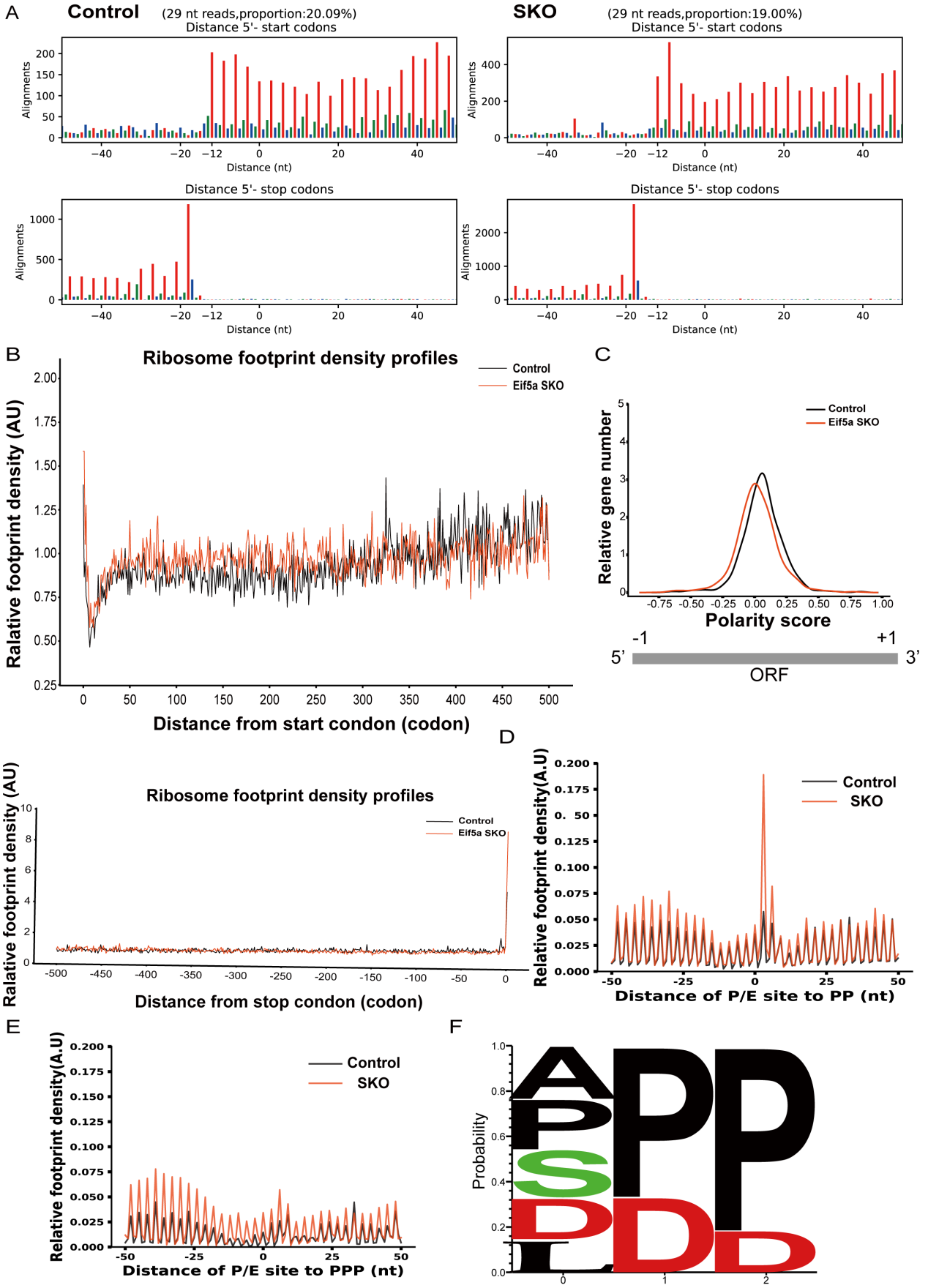
**

**Figure S4** ***Eif5a* promotes global translation elongation and termination in round spermatids.**

1. The 29-nt of Conrol(left) and *Eif5a* SKO (right) RPF read distributions around the ribosomal P site. The P site positions are colored according to the frame.
2. Ribosome densities along the CDS regions. Up: read density after the start codon. Down: read density before the stop codon.
3. Distribution of polarity scores for relative genes from control and *Eif5a* SKO round spermatids are plotted.
4. Average ribosome occupancy centered at diproline motifs with the underlined Pro in the P site of the ribosome.
5. Similar to D, average plot centered at triproline motifs with underlined Pro in the E site of the ribosome.
6. The tri-AA motifs with enriched ribosome, reported by RiboMiner. 0,1,2 represents E, P, A site of a tri-AA motif, respectively.

**
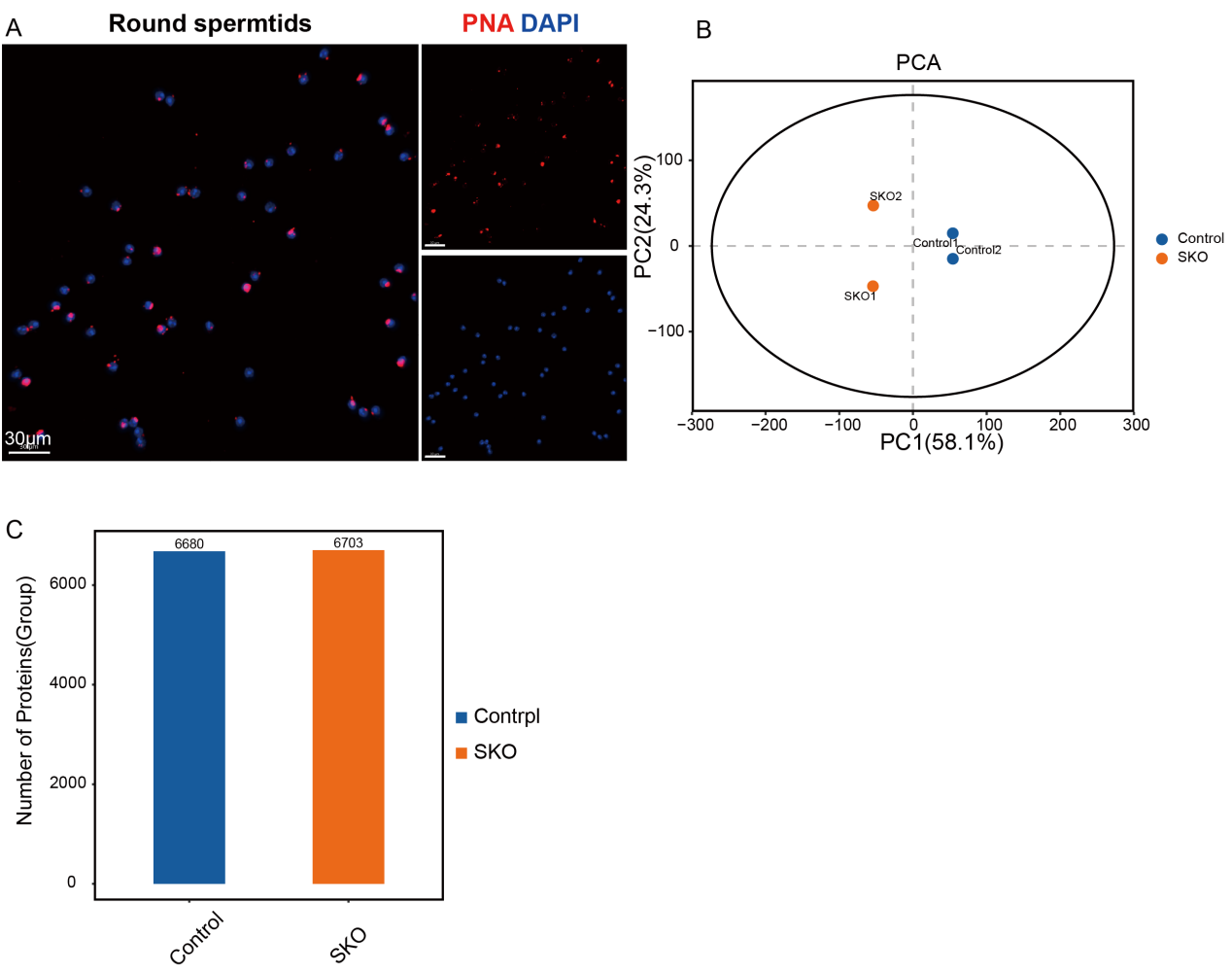
**

**Figure S5 Proteomic analyses of round spermatids from control and *Eif5a* SKO mice**

1. Identity confirmation of the round spermatids sorted by FASC.Scale bars represent 30 µm. Round spermatids stained with PNA and DAPI.
2. Principal component analysis (PCA) of proteins in round spermatids samples. control participants are represented in blue and *Eif5a* SKO in red.
3. Detected protein numbers of control and *Eif5a* SKO round spermatids.

**
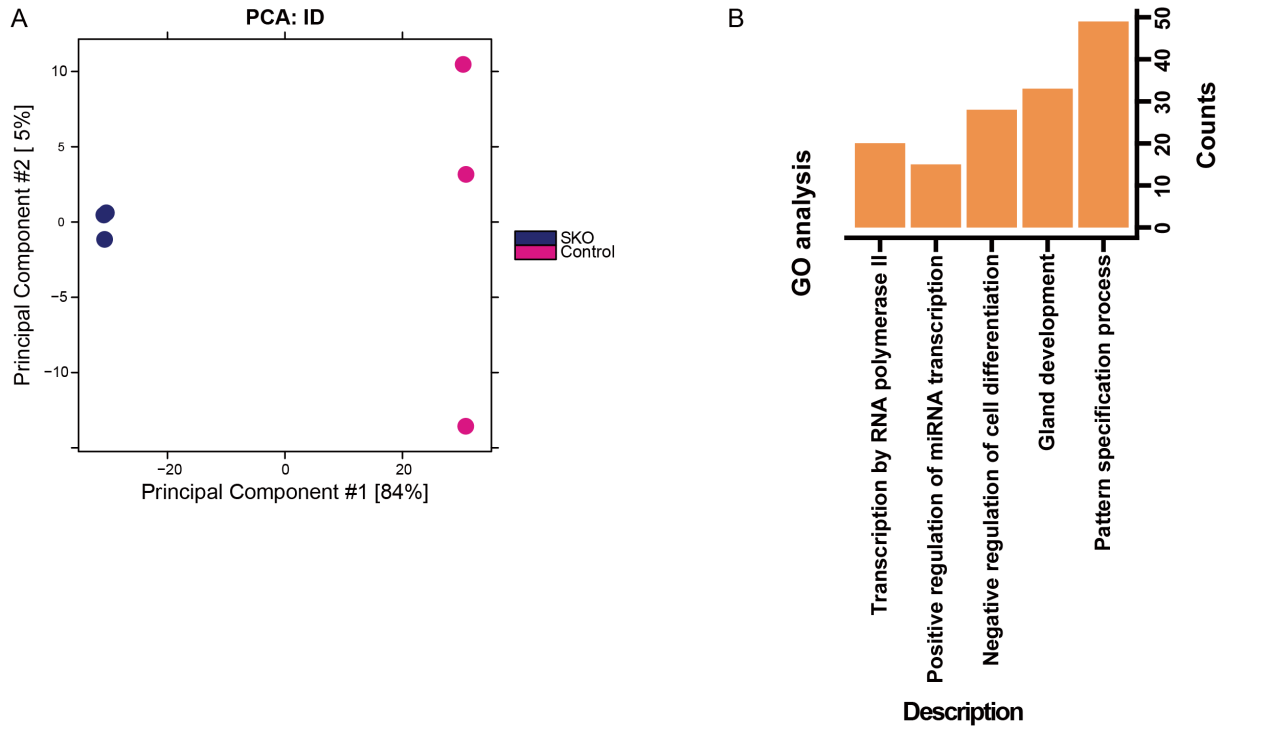
**

**Figure S6 ATAC-seq analyses of round spermatids from control and *Eif5a* SKO round spermatids.**

1. Principal component analysis (PCA) of ATAC-seq of control and *Eif5a* SKO round spermatids samples. control participants are represented in blue and *Eif5a* SKO in red.
2. GO analysis for transcription factor genes of *Eif5a* SKO gain motif.


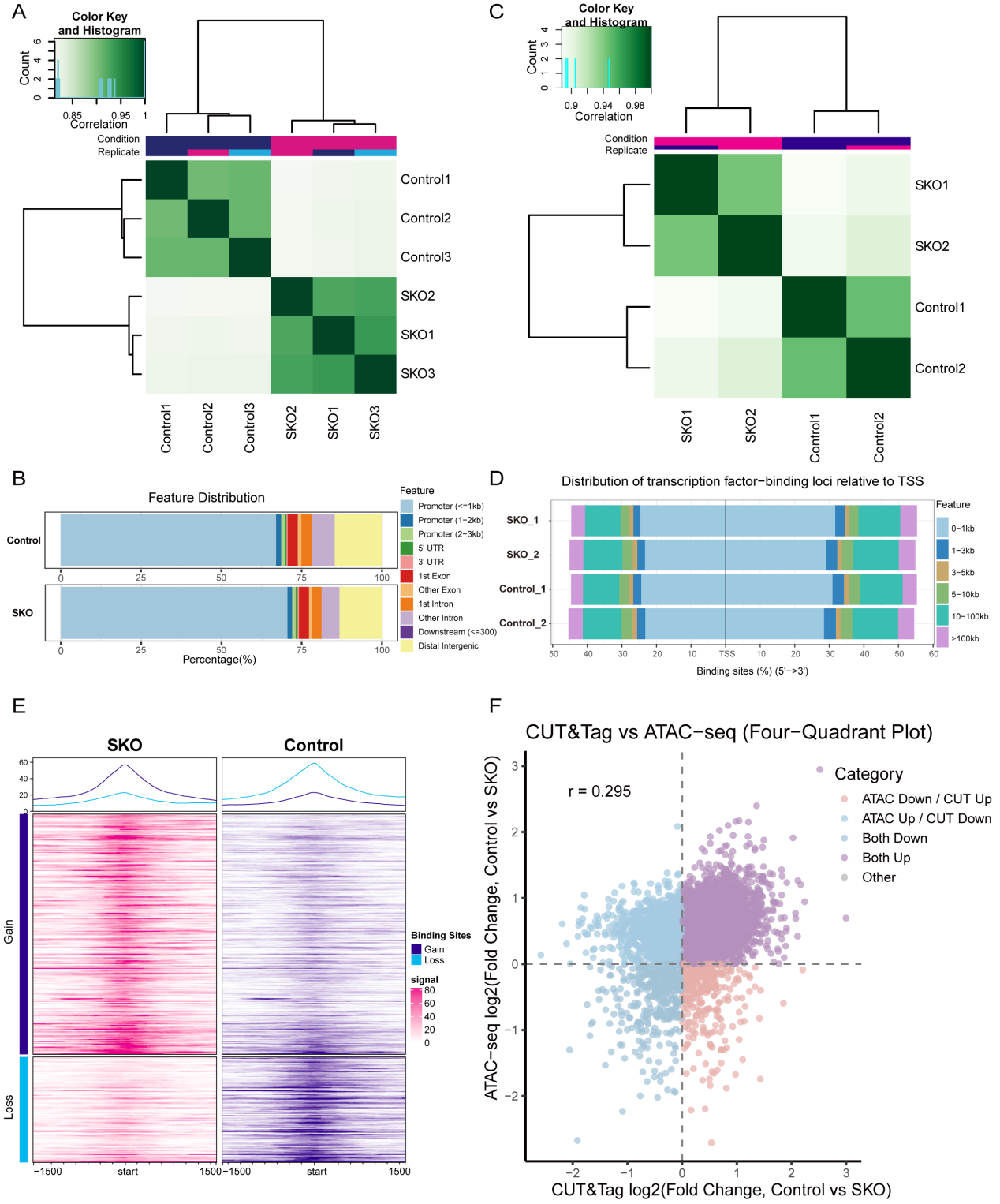


**Figure S7 Complementary ATAC-seq and CUT&Tag profiling identifies coordinated chromatin remodeling in eIF5A-deficient spermatids.**

1. Correlation heatmap using ATAC-seq affinity data shows that the fulvestrant treatment is significant enough to result in sample grouping. Numbers indicate technical replicates. Correlation (color key) indicates the Pearson correlation coefficient.
2. Genomic location annotations of ATAC-seq peaks. Distribution of genomic locations of the called peaks for control and *Eif5a* SKO libraries (with duplicates merged). Annotations were provided by ChIPseeker.
3. Sample correlation heatmap of CUT&Tag data, verifying data quality and replicate consistency.
4. Distribution profile of H3K4me3 peaks centered on transcription start sites (TSS).
5. Heatmap of H3K4me3 signals at differential binding regions, showing distinct patterns between control and SKO.
6. Four-quadrant scatter plots comparing the log₂ fold changes of all (left) or significant (right) peaks from CUT&Tag (x-axis) and ATAC-seq (y-axis), demonstrating a positive concordance between the two modalities.

**
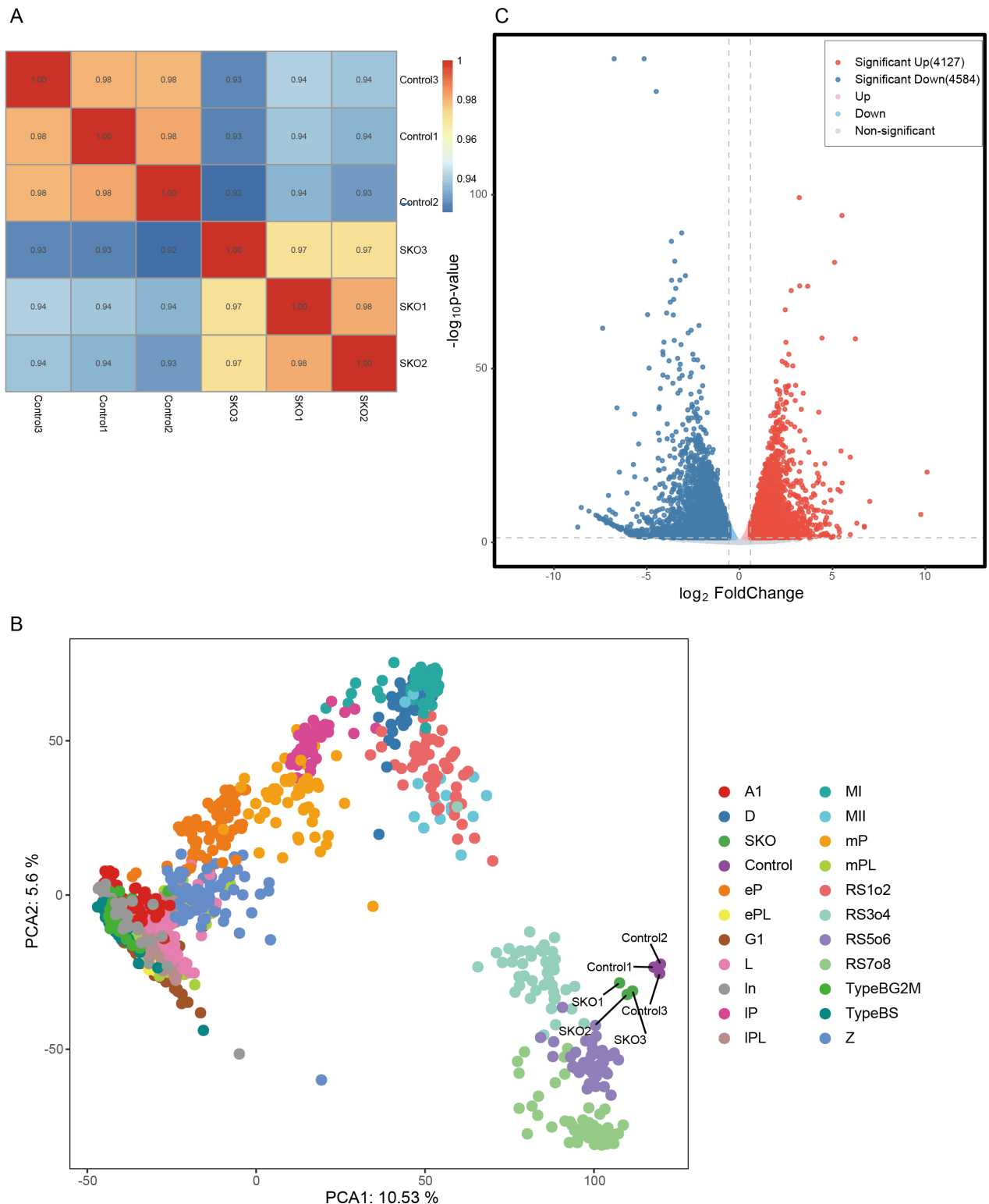
**

**Figure S8 Smart-seq2 analyses of round spermatids from control and *Eif5a* SKO mice.**

1. The heatmap displays the Pearson correlation between control and *Eif5a* SKO samples obtained through the Smart-seq2 technique.
2. Principal Component Analysis (PCA) of control, Eif5a SKO and different types of germ cells in the testis. Individual PCAs were combined using Procrustes transformation as previously described (Chen Y, et al. Cell Res. 2018).
3. Volcano plot showing differential genes in Eif5a SKO identified by Smart-seq2 analyses.

**Table S1 Primer sequences for quantitative RT-qPCR analysis of acrosome-and microtubule-related genes in round spermtids.**

| **Genes** | **Primer sequences( 5’-3’)** |
| --- | --- |
| SPACA3-F | GTTTTACGAGTGATGGGGCA |
| SPACA3-R | GGCTCCTAGCTTCCATGCG |
| spaca9-F | GAGCAGGTGCAGAGCTACAT |
| spaca9-R | CTTCGTGCGGATACTTGGCT |
| Spata1-F | AAATGGGTGCACCTTTCCGA |
| Spata1-R | CGCCAATGCTCCCTGTTCAT |
| Cbs7-F | GCTGAAGATGGATCGCCCAA |
| Cbs7-R | AGGAATGGTGTTGTCAGCCC |
| Dynlt3-F | TGGACTGCAAGCATAGTGGAA |
| Dynlt3-R | GTGAAATCCATACGGGCTCCT |
| Ly6k-F | TTGTTGGCCGTCACACGAAT |
| Ly6k-R | GTGGCCCCTCACCATTACAT |
| Tubb5-F | GATCGGTGCTAAGTTCTGGGA |
| Tubb5-R | AGGGACATACTTGCCACCTGT |
| Spata19-F | ATCTTTGCCCGGAAAACCGTA |
| Spata19-R | AGCTTCGCTTTCTTCAACTTCA |
| Lamp2-F | GGCTAATGGCTCAGCTTTCAAC |
| Lamp2-R | CGCTATGGGCACAAGGAAGT |
| Ccdc169-F | GGTTGTGGGCTTACAGGATGG |
| Ccdc169-R | GCTGCACAACATCCCTGGTTTC |

**Table S2 Primer sequences for TPRT-qPCR analysis in round spermtids.**

| **Genes** | **Primer sequences( 5’-3’)** |
| --- | --- |
| Cbx5-A1 | agcggataacaatttcacacaggcaggaaacagctatgacTCTTGGTCTTCTTTCC |
| Cbx5-BF1 | TCAACAGGCACACGACATGGG |
| Hmgb2-A3 | agcggataacaatttcacacaggcaggaaacagctatgacGTTGGGGTCACCCTTG |
| Hmgb2-BF3 | ACGCCGCGCCGTCGTCATGGG |
| Bscl2-A4 | agcggataacaatttcacacaggcaggaaacagctatgacGTACAGAGTGGCTAGG |
| Bscl2-BF4 | GCTGCGCGGCGGCACCATGTT |
| Gapdh-A14 | agcggataacaatttcacacaggcaggaaacagctatgacACCCCGGGGTAAGGGC |
| Gapdh-BF14 | TCAACCCTTAAGAGGGATGCT |
| Common BR | agcggataacaatttcacacagg |

**Table S3 Primer sequences for genotyping mouse genotypes.**

| **Genes** | **Primer sequences( 5’-3’)** |
| --- | --- |
| Eif5a-flox-F3 | GTTTCCTGTACCAATGACTGGCT |
| Eif5a-flox-R5 | CACAATGACATCTAAAGTTGTGGCT |
| Eif5a-(-)-F5 | CTACTTTCTCTCCCTTGACTGCAT |
| Eif5a-(-)-F6 | GAAATGCTGAGACTTCATAGTAGCC |
| Eif5a-(-)-R7 | GCTGGAATACCTACCCACTGATAA |
| *Stra8-GFPCre*–F | ACTCCAAGCACTGGGCAGAA |
| *Stra8-GFPCre*-R1 | GCCACCATAGCAGCATCAAA |
| *Stra8-GFPCre*-R2 | CGTTTACGTCGCCGTCCAG |
